# Diet-Induced Non-alcoholic Fatty Liver Disease and Associated Gut Dysbiosis Are Exacerbated by Oral Infection

**DOI:** 10.3389/froh.2021.784448

**Published:** 2022-01-24

**Authors:** Alexandra M. Simas, Carolyn D. Kramer, Caroline A. Genco

**Affiliations:** ^1^Gerald J. and Dorothy R. Friedman School of Nutrition and Science Policy, Graduate Program in Biochemical and Molecular Nutrition, Tufts University, Boston, MA, United States; ^2^Department of Immunology, Tufts University School of Medicine, Boston, MA, United States; ^3^Graduate Program in Immunology, Graduate School of Biomedical Sciences, Tufts University, Boston, MA, United States; ^4^Graduate Program in Molecular Microbiology, Graduate School of Biomedical Sciences, Tufts University, Boston, MA, United States

**Keywords:** microbiota, NAFLD, anaerobe, periodontal infection, Western diet

## Abstract

Increasing evidence indicates that chronic inflammation due to periodontal disease is associated with progression of non-alcoholic fatty liver disease (NAFLD) caused by a Western diet. NAFLD has also been associated with oral infection with the etiological agent of periodontal disease, *Porphyromonas gingivalis*. *P. gingivalis* oral infection has been shown to induce cardiometabolic disease features including hepatic lipid accumulation while also leading to dysbiosis of the gut microbiome. However, the impact of *P. gingivalis* infection on the gut microbiota of mice with diet-induced NAFLD and the potential for those changes to mediate NAFLD progression has yet to be determined. In the current study, we have demonstrated that *P. gingivalis* infection induced sustained alterations of the gut microbiota composition and predicted functions, which was associated with the promotion of NAFLD in steatotic mice. Reduced abundance of short-chain fatty acid-producing microbiota was observed after both acute and chronic *P. gingivalis* infection. Collectively, our findings demonstrate that *P. gingivalis* infection produces a persistent change in the gut microbiota composition and predicted functions that promotes steatosis and metabolic disease.

## Introduction

Non-alcoholic fatty liver disease (NAFLD) is the most common liver condition in the Western world and increasingly necessitates liver transplantation [1, 2]. NAFLD comprises a spectrum of progressive liver damage pathologies defined by hepatic lipid accumulation (steatosis). In over 40% of subjects with NAFLD, hepatic steatosis eventually progresses to non-alcoholic steatohepatitis (NASH), a state of hepatic inflammation which can eventually lead to advanced fibrosis, cirrhosis, and even liver cancer [3–6]. NAFLD is strongly associated with type 2 diabetes mellitus in humans, and NASH patients commonly demonstrate hepatic insulin resistance [7]. A disruption to the homeostasis of the complex gut microbiota, or gut dysbiosis, and microbial metabolite fluctuations are now considered pathogenic factors in human NAFLD progression [8–11]. Lactate and short-chain fatty acids (SCFAs) are important metabolites produced by the gut microbiota and can directly promote host metabolism and suppress inflammation [12, 13]. Reduced production of certain SCFAs has been identified as an important pathogenic factor in NAFLD and some SCFA producers have been identified as potential therapeutic agents [14–17].

Oral infection with *Porphyromonas gingivalis*, the etiological agent of periodontal disease, is associated with NAFLD progression in humans and in mice [18–20]. *P. gingivalis* oral infection induces dysbiosis of the oral microbiota which has been implicated in oral inflammation and bone loss [21, 22]. We have recently demonstrated that *P. gingivalis* oral infection induces dysbiosis of the gut microbiome in atherosclerotic mice [23]. However, the effect of *P. gingivalis* on the gut microbiota of steatotic mice fed a Western diet (WD) and the potential for those changes to mediate NAFLD progression has yet to be uncovered. To elucidate mechanisms by which *P. gingivalis* could influence NAFLD progression, we examined hepatic inflammation and metabolic dysfunction induced by *P. gingivalis* oral infection in mice fed either a control diet (CD) or a steatosis-inducing high-fat, high-sucrose WD. We demonstrate for the first time that the exacerbation of diet-induced steatosis and glucose intolerance by *P. gingivalis* oral infection of mice fed a WD is associated with distinct alterations in the gut microbiota composition and predicted functions, characterized by reductions in community diversity and short-chain fatty acid (SCFA) producers.

## Results

### *P. gingivalis* Promotes Hepatic Lipid Accumulation

**Figure 1:**
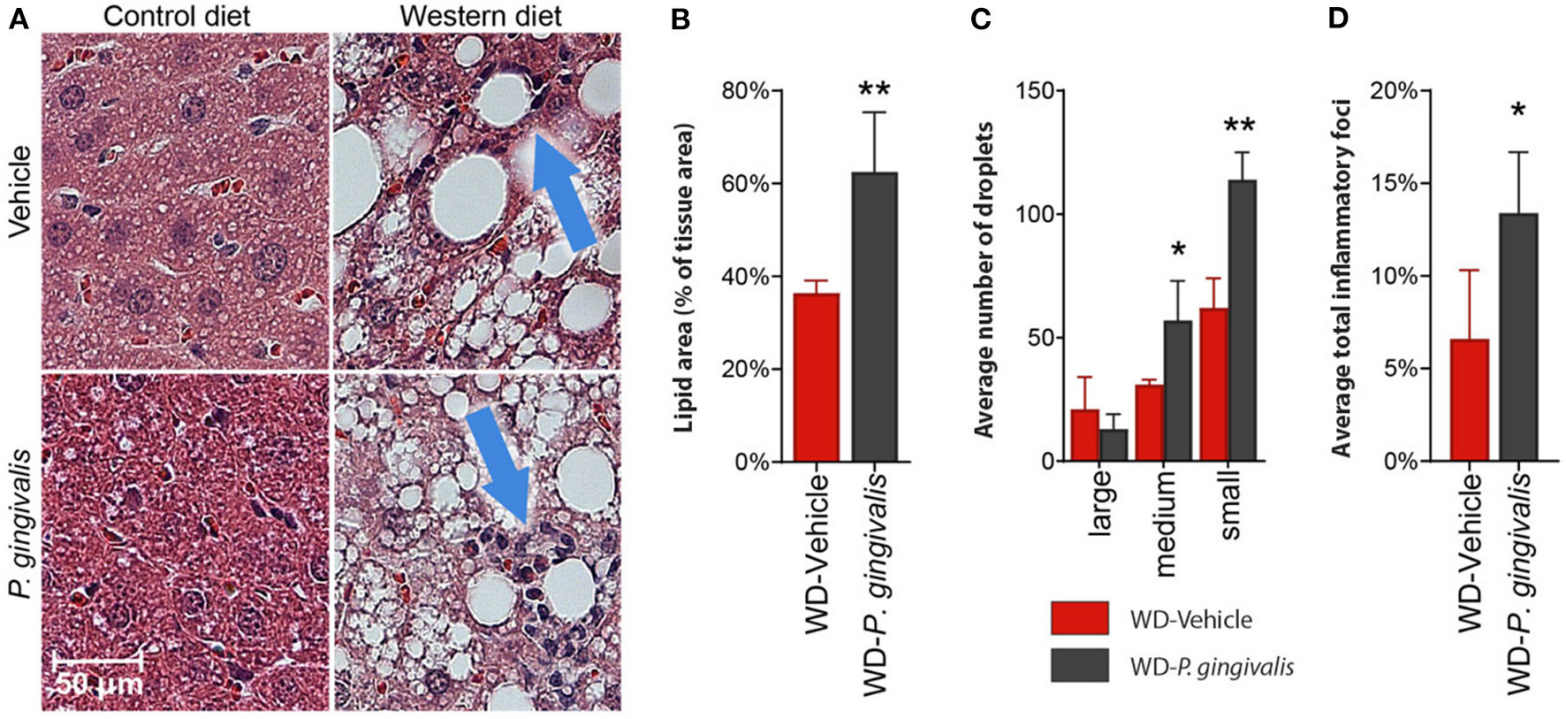
*P. gingivalis* infection exacerbates hepatic steatosis in mice fed a WD. **(A)** H&E stained, paraffin-embedded liver sections from control diet- or Western diet-fed mice that were either infected with *P. gingivalis* or treated with vehicle. Blue arrows indicate inflammatory foci. **(B)** Lipids quantified as lipid area as percent of total tissue area. **(C)** Lipid droplets binned by area. Red = vehicle-treated mice fed a WD, black = *P. gingivalis*-infected mice fed a WD. **(D)** Inflammatory foci quantified as foci per view. CD, control diet; WD, Western diet. Lipid droplets were only measured in the WD groups because there were so few in CD-fed mice. *p < 0.05, **p < 0.01 *P. gingivalis*-infected vs. vehicle-treated mice fed a WD. *n* = 5–8/group.

To evaluate the impact of *P. gingivalis* infection on WD-induced NAFLD, mice fed either a CD or a WD for 4 weeks were infected with *P. gingivalis* or treated with vehicle for 3 weeks and sacrificed after a total of 16 weeks (Supplementary Figure 1). To evaluate the influence of *P. gingivalis* infection on the progression of WD-induced NAFLD, steatosis and hepatic inflammation were evaluated by H&E staining of the liver (Figure 1A). WD feeding of vehicle-treated mice resulted in hepatic steatosis when compared with mice fed a CD (Figure 1A). *P. gingivalis* oral infection induced higher total lipid surface area in liver samples when compared with vehicle-treated mice fed a WD (Figure 1B). The increased steatosis due to *P. gingivalis* infection of mice fed a WD when compared with their vehicle-treated counterparts was characterized by a higher abundance of medium and small lipid droplets (Figure 1C). Steatosis was accompanied by an increase in the number of inflammatory foci as compared with vehicle-treated, WD-fed mice (Blue arrows in Figures 1A,D). These results indicate that *P. gingivalis* oral infection further disrupts lipid metabolism in the livers of steatotic mice. Minimal staining was observed in the livers of vehicle-treated and *P. gingivalis*-infected mice fed a WD stained with Picrosirius Red (collagen, reticulin) or antibodies against CD4^+^ T cells and F4/80 (macrophages) (data not shown).

### *P. gingivalis* Infection Impairs Glucose Tolerance

**Figure 2:**
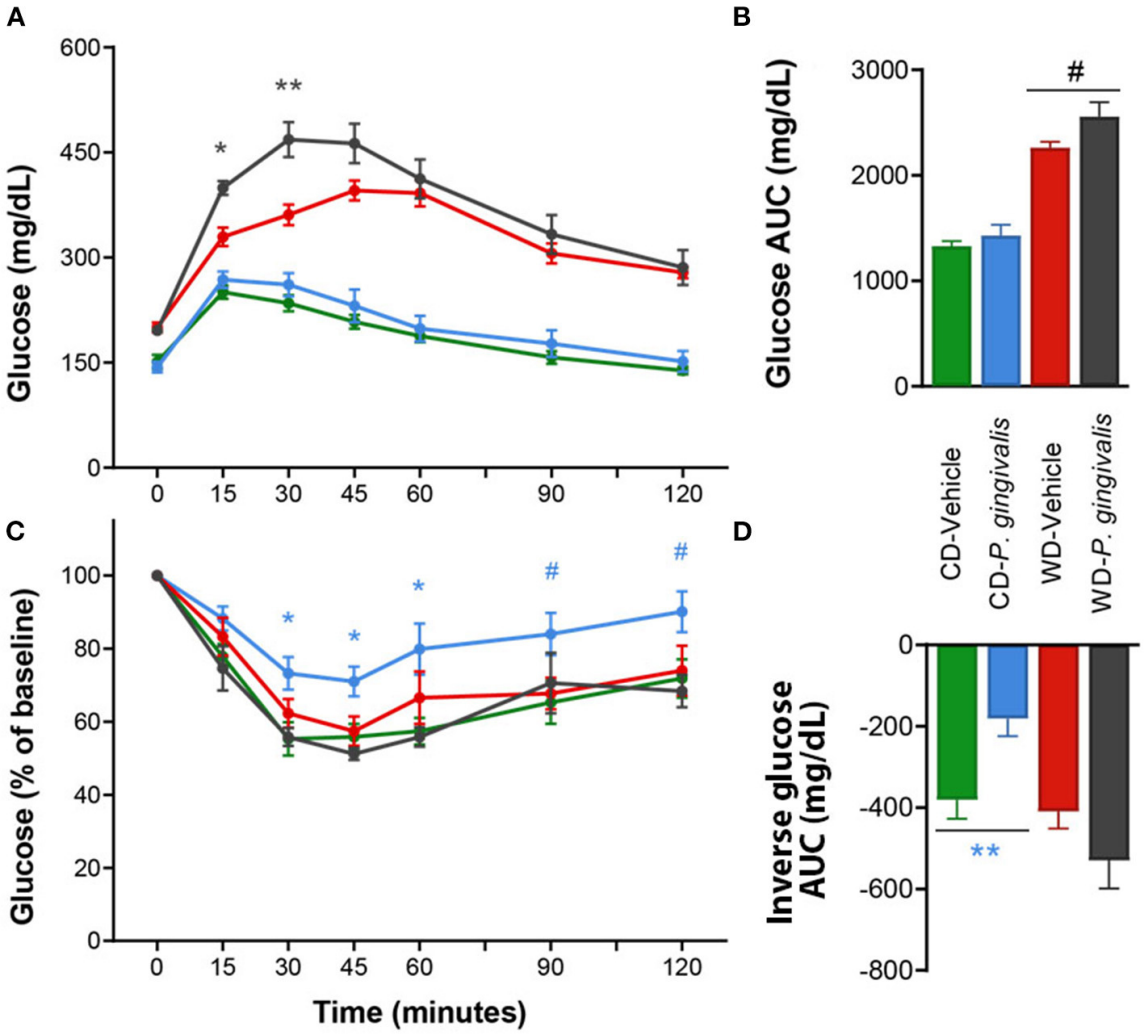
Altered glucose tolerance following *P. gingivalis* infection. Intraperitoneal glucose tolerance test over time **(A)** and as total glucose area under the curve (AUC) **(B)** during the 120-min test. Intraperitoneal insulin tolerance test over time **(C)** and as total glucose AUC **(D)**. Repeated measures or one-way ANOVA with a Bonferroni *post-hoc* performed as appropriate. CD, control diet; WD, Western diet. Green = vehicle-treated mice fed a CD, blue = *P. gingivalis*-infected mice fed a CD; red = vehicle-treated mice fed a WD, black = *P. gingivalis*-infected mice fed a WD. Asterisks represent effect of *P. gingivalis*-infected vs. diet-matched, vehicle-treated group. #*p* = 0.10, *p < 0.05, **p < 0.01. *n* = 5–8/group.

Periodontal disease has been tightly linked to insulin resistance in obese and non-obese human studies [24]. Insulin resistance correlates strongly with NAFLD severity and is believed to play a crucial role in the pathogenesis of NAFLD in humans [25, 26]. To evaluate the effect of *P. gingivalis* on glucose metabolism, we measured fasting blood glucose levels, glucose tolerance, and insulin tolerance. *P. gingivalis* infection of mice fed either a CD or a WD did not influence basal fasting blood glucose levels, though WD feeding of vehicle-treated mice did when compared with vehicle-treated mice fed a CD (Supplementary Table 1). *P. gingivalis* infection resulted in impaired glucose tolerance in mice fed a WD when compared with vehicle-treated mice fed a WD (Figures 2A,B). The impairment of glucose tolerance in *P. gingivalis*-infected mice was not associated with a difference in insulin sensitivity (Figures 2C,D). *P. gingivalis* infection reduced the insulin sensitivity of mice fed a CD (Figures 2C,D). These results indicate that *P. gingivalis* infection exacerbates glucose intolerance experienced by mice fed a WD.

### *P. gingivalis* Alters Host Metabolic and Inflammatory Gene Expression

**Figure 3:**
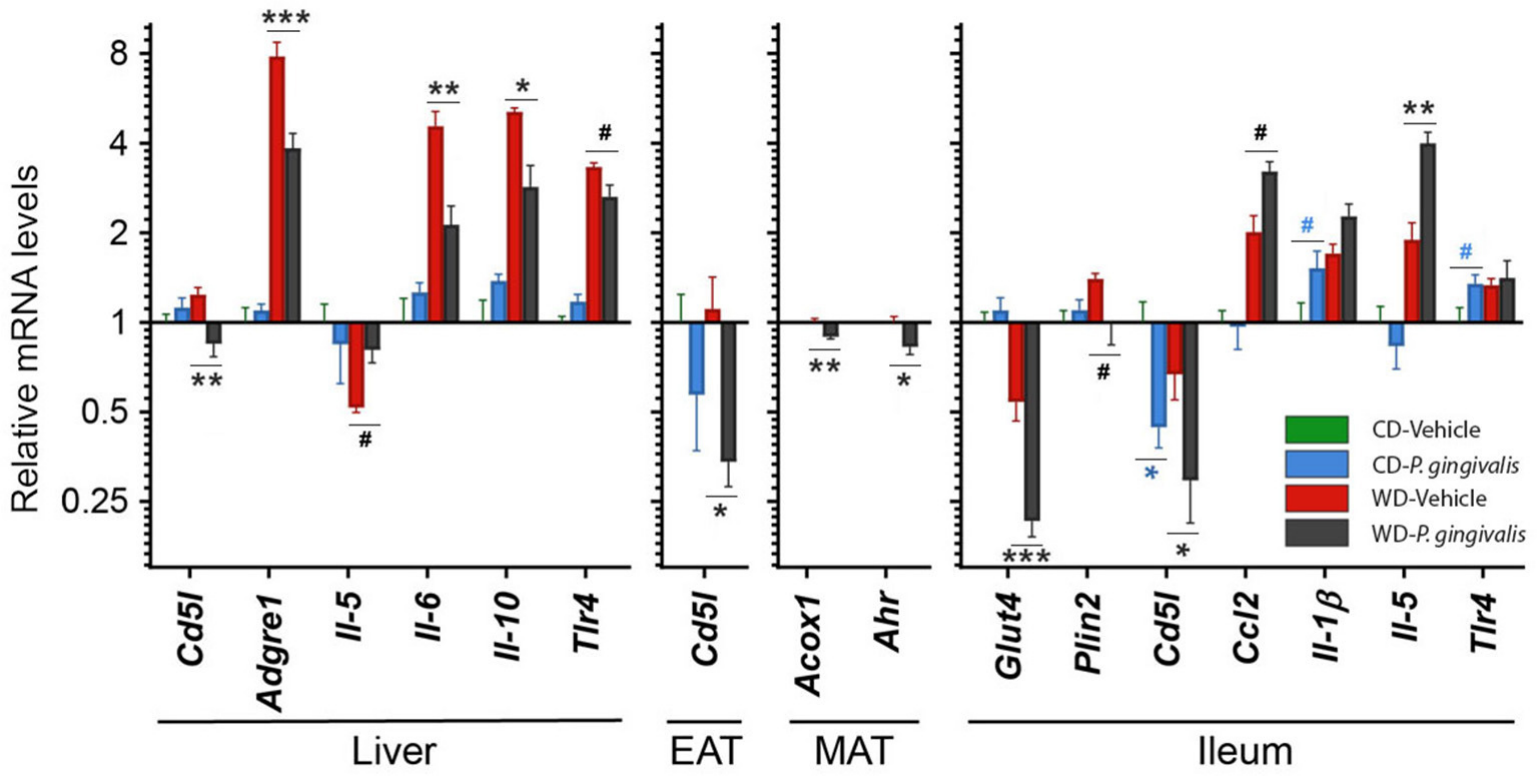
*P. gingivalis* infection promotes intestinal and hepatic inflammation in mice fed a Western diet. Expression profiles obtained from the liver, mesenteric adipose tissue or MAT, epididymal adipose tissue or EAT, and ileum. Gene expression, from mice fed a CD or a WD and either infected with *P. gingivalis* or treated with vehicle, was normalized relative to the baseline group (vehicle-treated mice fed a CD) and reported on a log_2_ scale as mean ± SEM. CD, control diet; WD, Western diet. Green = vehicle-treated mice fed a CD, blue = *P. gingivalis*-infected mice fed a CD; red = vehicle-treated mice fed a WD, black = *P. gingivalis*-infected mice fed a WD. Significance measured by a one-way ANOVA with a *post-hoc* Tukey test. Asterisks represent significant difference between *P. gingivalis*- vs. vehicle-treated mice fed a CD (blue asterisks) or fed a WD (gray asterisks). (#*p* < 0.1), *p < 0.05, **p < 0.01, ***p < 0.001. Genes with p>0.1 are reported in Supplementary Material. *n* = 5–8/group.

To identify host processes involved with *P. gingivalis*-induced steatosis and insulin resistance, we measured mRNA expression levels of genes associated with metabolism and inflammation in the liver, adipose tissue, and ileum of *P. gingivalis*-infected mice (Figure 3; see all gene expression results in Supplementary Table 2 online). Vehicle-treated mice fed a WD experienced increased expression of genes involved in pro-inflammatory signaling, macrophage recruitment, and bacterial sensing when compared with vehicle-treated mice fed a CD (Figure 3; Supplementary Table 2). *P. gingivalis* infection partially reversed many of the effects of WD feeding. In contrast to vehicle-treated, WD-fed mice, *P. gingivalis*-infected mice fed a WD experienced increased hepatic expression of the pro-inflammatory cytokine *Il-5* and reduced expression of the cytokines *Il-6* and *Il-10*, macrophage marker *Adgre1, Tlr4*, as well as *Cd5l*, which controls apoptosis and immune cell differentiation via regulating lipolysis (Figure 3). Together, these findings suggest that *P. gingivalis* infection suppresses some WD-induced inflammation-associated pathways while also inducing other inflammatory signaling pathways.

WD feeding increased the masses of all adipose tissue depots in vehicle-treated mice compared with their CD-fed counterparts (Supplementary Table 1). Mice fed a WD demonstrated an epididymal adipose tissue gene expression profile characterized by reduced insulin sensitivity and lipid oxidation but increased lipid accumulation, inflammation, macrophage recruitment, and adipose tissue remodeling (Supplementary Table 2). *P. gingivalis* infection resulted in an increase in the weight of the metabolically active mesenteric adipose tissue of mice fed a WD as compared with mice which were vehicle-treated (Supplementary Table 1). The increase in mesenteric adipose tissue weight corresponded with decreased expression of the lipid oxidation gene *Acox1* and aryl hydrocarbon receptor (*Ahr*), a highly central regulatory gene involved in processes including antimicrobial peptide production, detoxification, and cell metabolism (Figure 3). The effect of *P. gingivalis* on the epididymal adipose tissue of WD-fed mice was limited to suppressed *Cd5l* expression when compared to vehicle-treated mice fed a WD (Figure 3).

To evaluate intestinal inflammation, we examined gene expression in the ileum, a site highly active in generation of host-interactive metabolites and the primary site of lipid absorption [27, 28] (Figure 3). When compared with vehicle-treated mice fed a WD, *P. gingivalis* infection of WD-fed mice induced expression of genes associated with inflammation (*Ccl2, Il-5*) but suppressed expression of genes associated with glucose transport and lipid storage (*Glut4, Plin2*) (Supplementary Table 2; Figure 3). In mice fed either a WD or a CD, *P. gingivalis* infection suppressed ileal *Cd5l* when compared with their vehicle-treated counterparts (Supplementary Table 2; Figure 3). WD feeding of vehicle-treated mice induced expression of genes involved in metabolic regulation, lipid storage, and inflammation and reduced expression of the glucose-sensitive transporter gene *Glut4* when compared with vehicle-treated mice fed a CD (Supplementary Table 2; Figure 3).

### *P. gingivalis* Infection of Mice Fed a Western Diet Alters Community Diversity

Structural disruption of gut microbiota and associated inflammation are considered important etiological factors in diet-induced insulin resistance and steatosis [29]. Since *P. gingivalis* infection exacerbated diet-induced steatosis, we next investigated whether *P. gingivalis* had an additional impact on the dysbiotic gut microbiota of mice fed a WD by performing 16S rRNA gene sequencing of the cecal contents collected from mice fed a WD or a CD and either infected with *P. gingivalis* or treated with vehicle. A total of 281 amplicon sequence variants or ASVs were identified from 5,994,592 total reads assigned to 490 non-chimeric sequences and filtered by presence in more than 20% of samples (Supplementary Figure 2).

**Figure 4:**
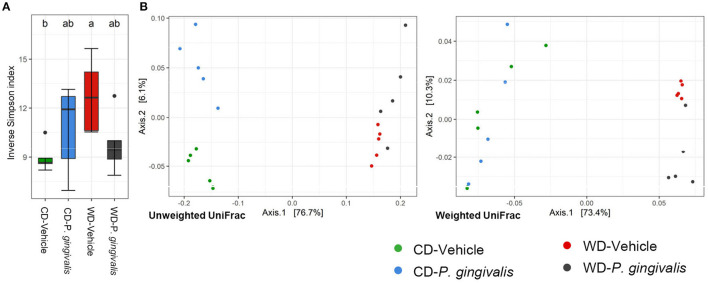
Chronic *P. gingivalis* infection alters microbial community abundance of mice fed a Western diet. Results of 16S rRNA gene sequencing of the cecal microbiota. **(A)** Inverse Simpson index measuring alpha diversity. Groups with different letters (a, b) are significantly different. **(B)** Unweighted and weighted UniFrac measurement of beta diversity with significance measured by PERMANOVA. Unweighted: all groups: *R*^2^ = 0.7332, *p* < 0.001; *P. gingivalis* vs. vehicle-treated mice fed a WD: *R*^2^ =0.1743, *p* = 0.169; *P. gingivalis* vs. vehicle-treated mice fed a CD: *R*^2^ =0.3505, *p* = 0.026. Weighted: *R*^2^ = 0.7332, *p* < 0.001; *P. gingivalis* vs. vehicle-treated mice fed a WD: *R*^2^ =0.1743, *p* = 0.169; *P. gingivalis* vs. vehicle-treated mice fed a CD: *R*^2^ =0.3505, *p* = 0.026.

There was no significant difference in alpha diversity due to *P. gingivalis* infection of WD-fed mice or CD-fed mice when compared with their vehicle-treated counterparts (Figure 4A; Supplementary Figure 3). To determine the dissimilarity between the experimental groups' community membership and structure, beta diversity was assessed by unweighted and weighted UniFrac distances. These indices use phylogenetic relatedness and measure ASV presence or abundance, respectively [30]. Among mice fed a WD, the microbiota of vehicle-treated and *P. gingivalis*-infected mice were distinct from one another when assessed for ASV abundance rather than ASV presence (Figure 4B; Supplementary Figure 4). This was also evident when the microbiota samples were measured by Euclidean distance of sample relatedness; within each diet-specific cluster, the microbiota of *P. gingivalis*-infected mice clustered separately from the microbiota of vehicle-treated and untreated mice (Supplementary Figure 4B). Variation between the microbiota of *P. gingivalis*-infected mice fed a WD was less than those of vehicle-treated mice fed a WD. WD feeding alone affected the gut microbiota resulting in increased alpha diversity as measured by the Shannon index (Supplementary Table 3), which summarizes both taxonomic richness and evenness, and caused diet-specific clustering of the microbiota from the four experimental groups by both weighted and unweighted UniFrac (Figure 4B; Supplementary Figure 4). In contrast to WD-fed mice, *P. gingivalis* infection of mice fed a CD was associated with distinct community presence but not abundance when compared with vehicle-treated mice fed a CD (Figure 4B; Supplementary Figure 4).

### Chronic *P. gingivalis* Infection Alters Gut Microbiota Composition and Functional Pathways

**Figure 5:**
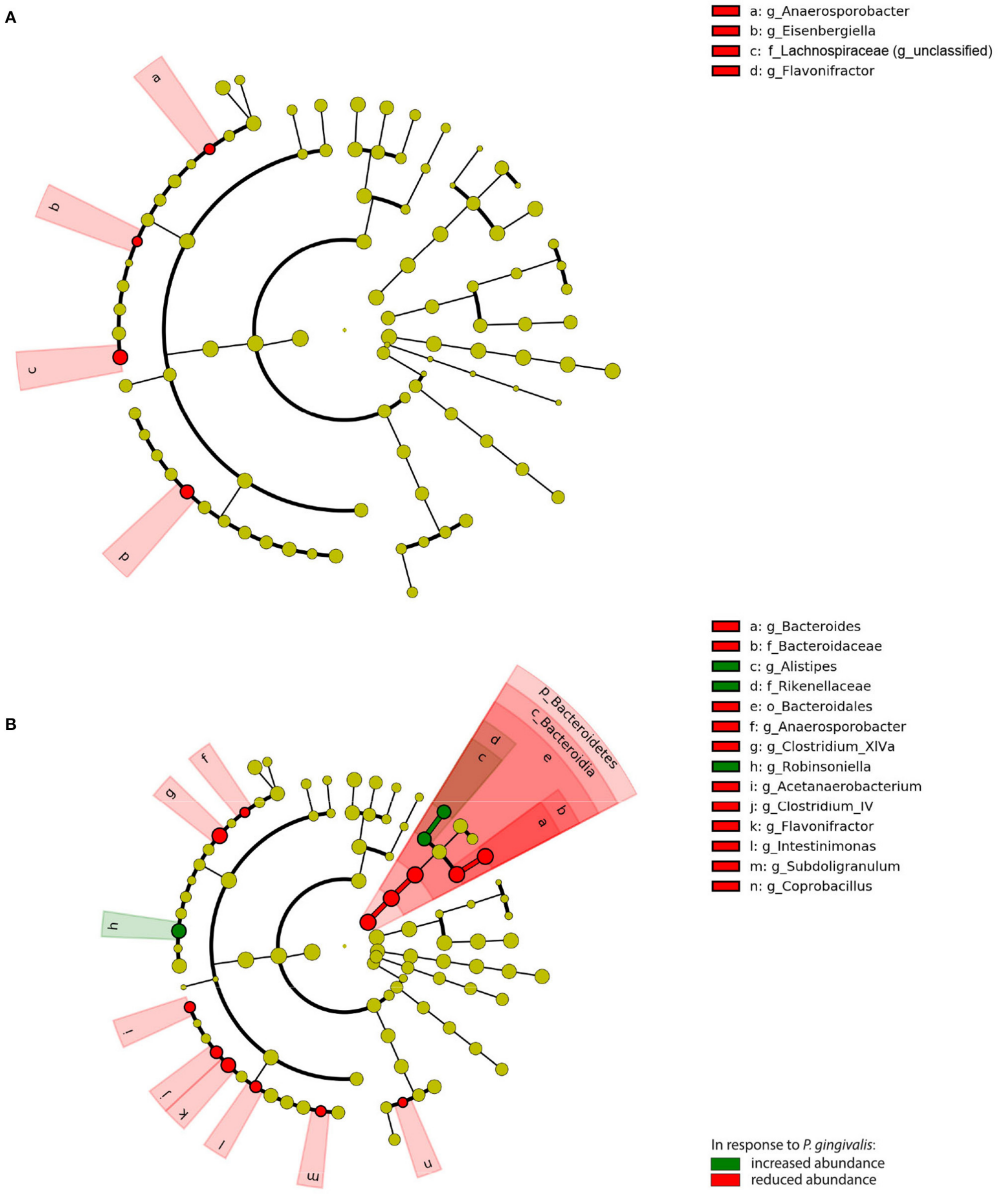
Chronic *P. gingivalis* infection persistently disrupts microbiota composition in mice fed a Western diet. Linear discriminant analysis (LDA) effect size (LEfSe) analysis of predicted taxonomy from 16S rRNA gene sequencing of the cecal microbiota. **(A)** *P. gingivalis* vs. vehicle-treated mice fed a WD. **(B)** *P. gingivalis* vs. vehicle-treated mice fed a CD. LDA > 2.0, α < 0.05. For all comparisons, green indicates increased abundance while red indicates reduced abundance in response to *P. gingivalis*.

To evaluate the significance of *P. gingivalis*'s influence on the gut microbiota, ASVs were pooled by their taxonomic assignment (Supplementary Figure 5) and differences in the taxa were measured using linear discriminant analysis effect size (LEfSe). When compared with vehicle-treated mice fed a WD, the gut microbiota of *P. gingivalis*-infected mice fed a WD demonstrated reductions in the abundance of unclassified members of the family Lachnospiraceae and the genera *Anaerosporobacter*, the flavonoid degrader *Flavonifractor*, and *Eisenbergella* (LDA > 2.0, α < 0.05) (Figure 5A). WD feeding of vehicle-treated mice produced expected differences in the gut microbiota composition when compared with vehicle-treated mice fed a CD (LDA > 2.0, α < 0.05) (Supplementary Figures 5, 6).

*P. gingivalis* infection induced more changes in the composition of the gut microbiota of mice fed a CD (Figure 5B; Supplementary Figure 5). The phylum Bacteroidetes was decreased in the ceca of *P. gingivalis*-infected mice fed a CD, primarily due to the genus *Bacteroides*, though the Bacteroidetes genus *Alistipes* was increased when compared with vehicle-treated, CD-fed mice. The effect of *P. gingivalis* on the gut microbiota of CD-fed mice was also characterized by increased abundance of the Firmicutes genus *Robinsoniella* and decreases in the genera including *Flavonifractor*, the lactate-producing *Coprobacillus*, and the butyrate-producing *Clostridium* cluster XIVa, a key resident of the colonic mucous layer.

**Figure 6:**
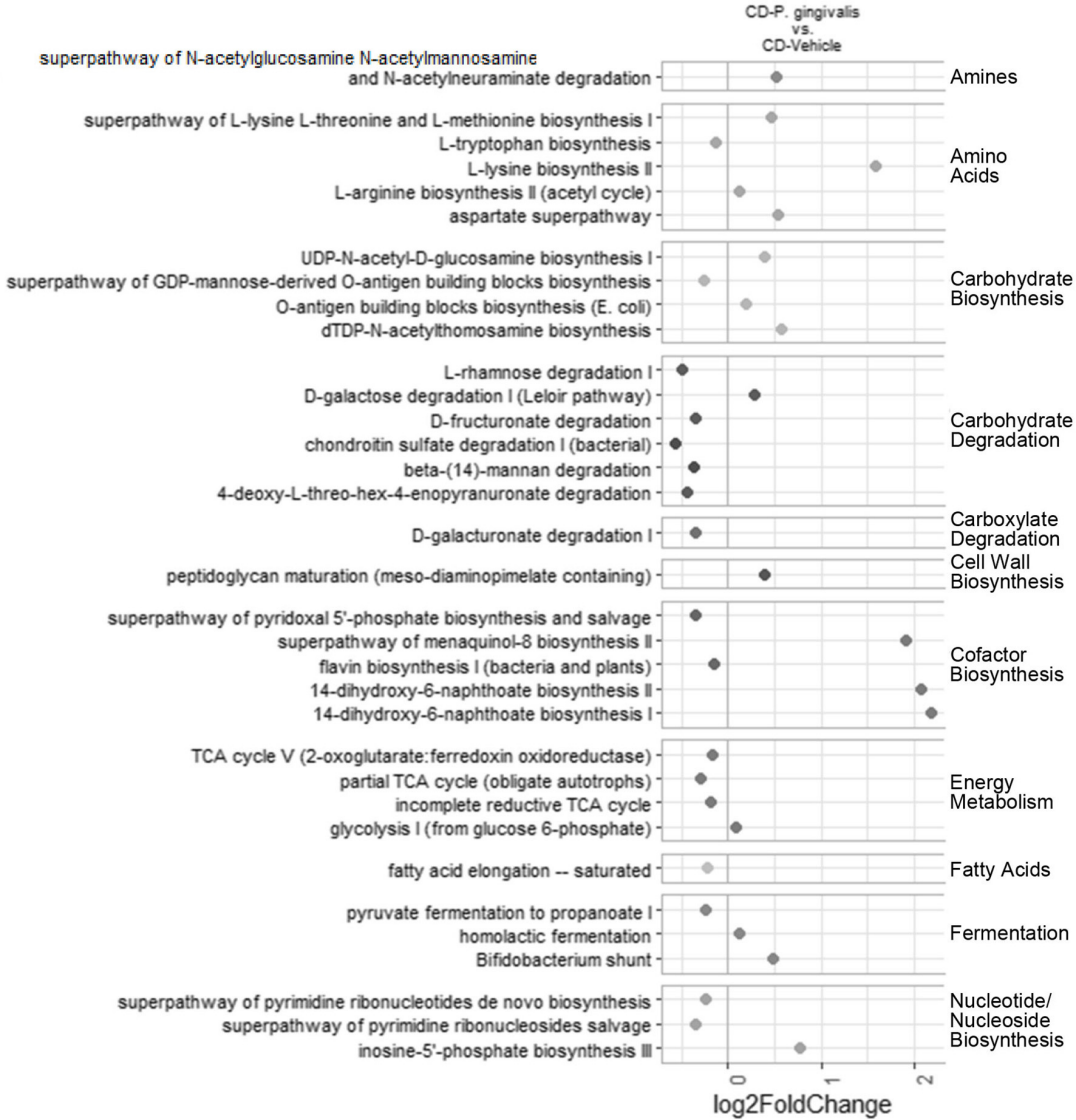
Chronic *P. gingivalis* infection persistently disrupts microbiota function in mice fed a control diet. Predicted pathways expected to be differentially abundant due to *P. gingivalis* infection of mice fed a CD. Significance assessed by DESeq2 with an adjusted *p*-value < 0.05.

To evaluate the potential functional significance of the observed microbiota changes, PICRUSt2 was used to predict metagenome content and contribution of the metagenome to MetaCyc pathways. No pathways were differentially abundant (adjusted *p*-value < 0.01 by DESeq2) in their inferred abundance when comparing *P. gingivalis*-infected, WD-fed mice with vehicle-treated, WD-fed mice. Comparing vehicle-treated, WD-fed mice with their CD-fed counterparts substantially changed the predicted metagenome content of the cecal microbiota (Supplementary Figure 7). The gut microbiota of *P. gingivalis*-infected mice fed a CD demonstrated predicted enrichment of multiple functions (Figure 6; Supplementary Figure 7). The largest fold differences included enrichment of some vitamin K and L-Lysine biosynthesis pathways and reduction of some carbohydrate degradation pathways. *P. gingivalis* infection of CD-fed mice was also associated with increased predicted potential of the *Bifidobacterium* shunt, which was in large part due to an increase in *Bifidobacterium* that did not quite achieve significance by LEfSe due to sample variability. Half of the pathways impacted by *P. gingivalis* infection in mice fed a CD were influenced by WD feeding in the same direction (Supplementary Figure 7). The only pathways affected in the opposite direction were the two naphthanoate and one menaquinol biosynthesis pathways, which were predicted to be enriched slightly in *P. gingivalis*-infected, CD-fed mice compared to vehicle-treated, CD-fed mice but highly reduced in vehicle-treated, WD-fed mice compared to vehicle-treated, CD-fed mice. Collectively, these results indicate that the combination of *P. gingivalis* and WD feeding results in the expression of distinct functional pathways by the gut microbiota.

### Correlation of Gut Microbiota Profile With Host Metabolism and Inflammation

**Figure 7:**
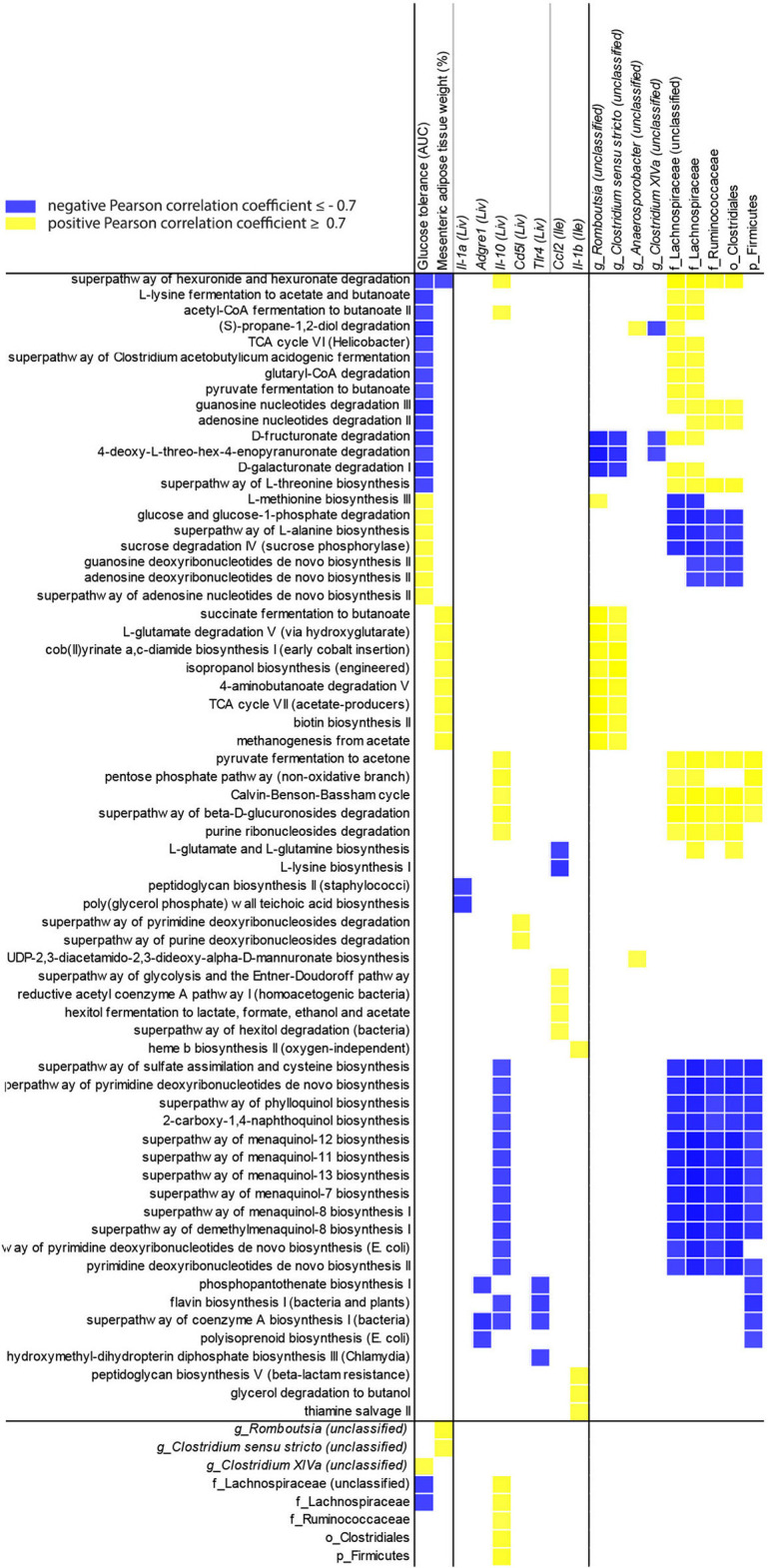
Changes in microbiota predicted functional pathways induced by *P. gingivalis* infection correlate with reduced glucose tolerance in mice fed a WD. Correlations between glucose tolerance test performance represented as the area under the curve, mesenteric adipose tissue weight, or gene expression and MetaCyc pathway abundance predicted from 16S rRNA gene sequencing of the cecal microbiota of WD-fed mice either infected with *P. gingivalis* or treated with vehicle. Host phenotype: Glucose tolerance = area under the curve during a glucose tolerance test (mg/dL); Mesenteric adipose tissue weight (%) = weight of mesenteric adipose tissue as a percent of total body weight. All pathways correlated with host phenotype or gene expression (cutoff = +/−0.7) were included, as well as any taxa they correlated with. Yellow = direct correlation (positive Pearson correlation coefficient), blue = direct correlation (negative Pearson correlation coefficient).

To evaluate the influence of the microbiota community on NAFLD, Pearson correlation coefficients for *P. gingivalis*- and vehicle-treated mice fed a WD (Figure 7) were calculated correlating taxa and MetaCyc pathways from the chronic timepoint with host gene expression and phenotype. In mice fed a WD, glucose tolerance and mesenteric adipose tissue weight were correlated with the differential abundance of a collection of pathways. This collection was enriched for cobalamin production and cobalamin-dependent pathways including (S)-propane-1,2-diol degradation, deoxyribonucleotide biosynthesis, methanogenesis, and multiple fermentation pathways. These pathways were primarily associated with the abundances of Lachnospiraceae, Ruminococcaceae, and Clostridiales. These findings suggest that elements of the microbiota composition and function are associated with glucose metabolism dysfunction and adiposity in mice fed a WD during chronic *P. gingivalis* infection.

### Acute *P. gingivalis* Infection Exacerbates Gut Dysbiosis Induced by a Western Diet

We previously demonstrated that *P. gingivalis* infection has distinct effects on host inflammatory and metabolic processes during acute vs. chronic infection [31]. However, associated changes to gut microbiota of mice fed a WD during acute vs. chronic *P. gingivalis* infection has not been investigated. To assess the impact of *P. gingivalis* acute infection on changes in the gut microbiota we collected feces 1 day after the final *P. gingivalis* exposure in mice fed either a WD or a CD (see Supplementary Figure 1). A total of 171 amplicon sequence variants or ASVs were identified from 771,769 total reads assigned to 392 non-chimeric sequences and filtered by presence in more than 20% of samples (Supplementary Figure 8).

**Figure 8:**
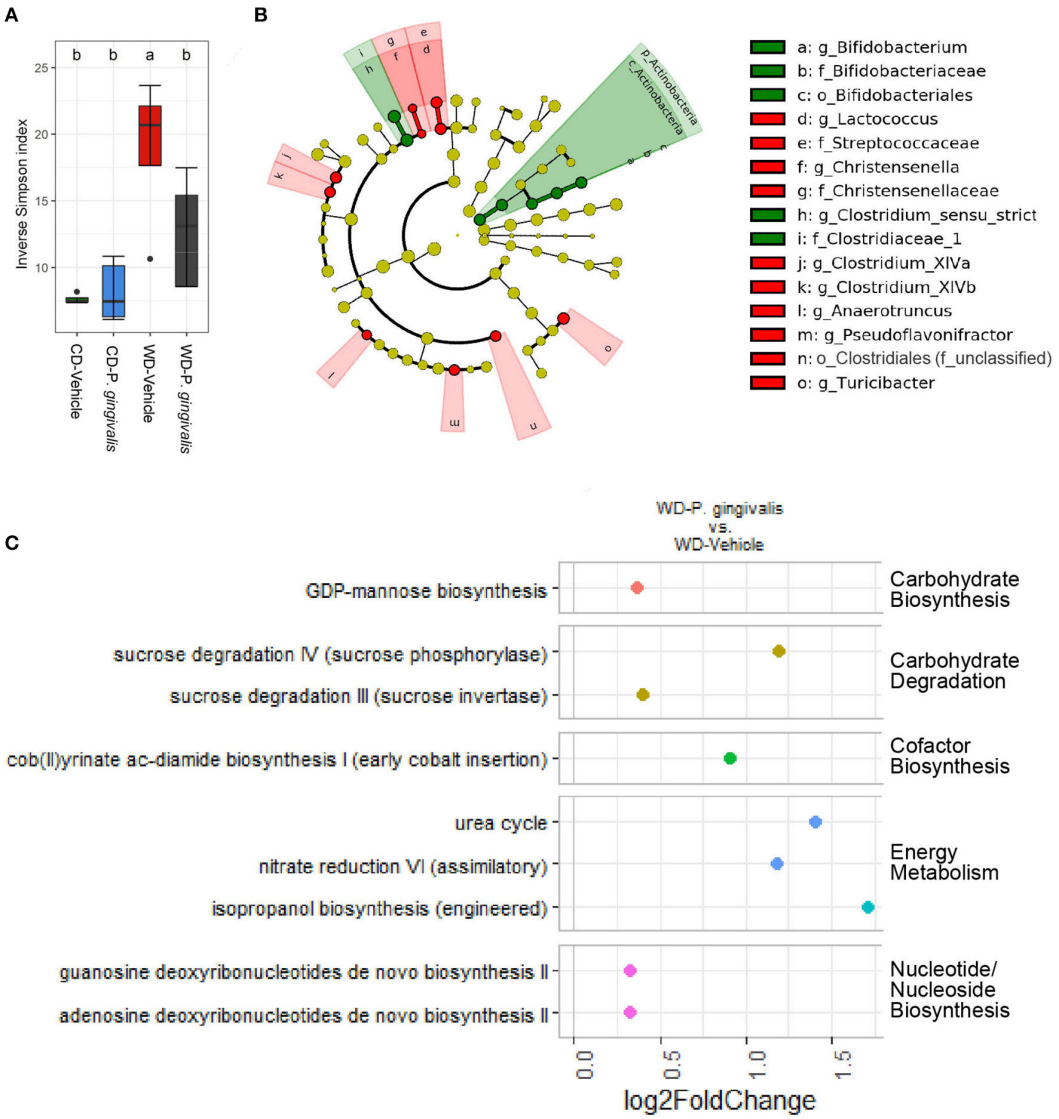
Acute *P. gingivalis* infection induces dysbiosis in mice fed a Western diet. Results of 16S rRNA gene sequencing of the fecal microbiota collected 1 day after the final *P. gingivalis* infection. **(A)** Inverse Simpson index measuring alpha diversity. Groups with different letters (a, b) are significantly different. **(B)** Linear discriminant analysis (LDA) effect size (LEfSe) analysis of the predicted taxonomy of *P. gingivalis* vs. vehicle-treated mice fed a WD during acute infection. LDA > 2.0, α < 0.05. Green indicates increased abundance while red indicates reduced abundance in response to *P. gingivalis*. **(C)** Predicted pathways expected to be differentially abundant when comparing the inferred cecal metagenome enrichment of *P. gingivalis*-infected, WD-fed mice and vehicle-treated, WD-fed mice. No significant differences were observed between *P. gingivalis*-infected, CD-fed mice and vehicle-treated, CD-fed mice. Significance assessed by DESeq2 with an adjusted *p*-value < 0.01.

Acute *P. gingivalis* infection reduced alpha diversity as measured by the Inverse Simpson index, which preferentially considers the most common species when evaluating both evenness and richness [32], reversing the effect of WD feeding (Figure 8A; Supplementary Figure 9). Acute *P. gingivalis* infection did not influence the beta diversity of the fecal microbiota collected from mice fed either a WD or a CD (Supplementary Figure 10). Acute infection of mice fed a WD resulted in increased abundance of the phylum Actinobacteria (due to the genus *Bifidobacterium*) and the genus *Clostridium sensu stricto*, which represents the truest Clostridium species [33], when compared with vehicle-treated mice fed a WD (Figure 8B; Supplementary Figure 11). Decreases of genera including *Turicibacter, Lactococcus, Christensenella*, the *Clostridium* clusters XIVa and XIVb, and *Anaerotruncus* were also observed (Figure 8B).

During *P. gingivalis* acute infection we observed substantial inferred enrichment of predicted pathways including the cob (II) yrinate ac-diamide biosynthesis pathway, the anaerobic pathway for the production of B vitamin and cofactor cobalamin, and the urea cycle (Figure 8C). WD feeding during the acute phase was associated with a predicted enrichment of fermentation pathways, altered ratios of energy metabolism and TCA cycle pathways, and was also associated with increased inferred urea cycle potential (Supplementary Figure 12). *P. gingivalis* acute infection did not significantly alter the gut microbiota of mice fed a CD at the taxonomic level. Our findings suggest that *P. gingivalis* oral infection minimally disrupted the gut microbiota of mice fed a CD. However, *P. gingivalis* infection immediately perturbs the already dysbiotic gut microbiota of mice fed a WD, leading to persistence of a more moderate form of dysbiosis characterized by reduced community diversity and increased variation between samples during chronic infection.

## Discussion

We demonstrate here that *P. gingivalis* oral infection disrupted the gut microbiota composition of mice fed a WD, resulting in functional changes associated with exacerbated NAFLD. Although *P. gingivalis* translocates to distant organs such as the liver, our observations support a role as a keystone pathogen, orchestrating inflammation through gut dysbiosis. The observed perturbation during acute infection persisted during chronic infection in a milder form and was characterized by reduced community diversity and increased variation of the dysbiotic microbiota of individually infected mice fed a WD. Variation among the gut microbiomes of genetically identical mice is known to confer susceptibility to diet-induced metabolic disease and play a causal role in the development of diet-induced hyperglycemia and hepatic steatosis [34–37].

Our findings indicate that *P. gingivalis* infection further impaired the glucose tolerance of mice experiencing diet-induced metabolic dysfunction. The differences in predicted enrichment of fermentation and carbohydrate degradation pathways during *P. gingivalis* infection of mice fed a WD may result from changes in the availability of energy sources. Glucose tolerance was associated with the predicted abundance of many MetaCyc pathways, suggesting a strong relationship between glucose metabolism and the dysbiotic gut microbiota of *P. gingivalis*-infected mice fed a WD. In contrast, hepatic steatosis did not correlate with the microbiota but was strongly associated with a gene expression profile primarily characterized by increased expression of the pro-fibrotic Th2 cytokine *ll-5* and the pro-inflammatory *Il-1*α. These results suggest a primary role for inflammation in the development of hepatic steatosis, which is consistent with observations that *P.gingivalis* translocates to the liver and its LPS causes excessive hepatic lipid accumulation through activated inflammatory responses [38, 39]. Increased hepatic expression of *Il-1*α*/*β has been observed in diet-induced NAFLD mouse models, and deficiency of either one is protective [40, 41].

The most consistent effect of *P. gingivalis* infection on gene expression across tissues was the suppression of *Cd5l* expression in mice fed either a WD or a CD. Expressed predominantly in macrophages and hepatocytes, CD5L regulates immune cell recruitment, polarization, and apoptosis [42]. CD5L enhances antimicrobial functions of macrophages; the suppressed *Cd5l* expression in the intestine may support a role for *P. gingivalis* infection in shaping the gut microbiome by affecting host antimicrobial functions [43]. Along with *Il-1*α and *ll-5* expression, hepatic expression of *Cd5l* and *Il-10* support the potential for imbalances in macrophage polarization and T-cell differentiation due to *P. gingivalis* infection in mice fed a WD [44]. For example, *Il-10* plays a key role in reprogramming macrophages from the pro-inflammatory M1 phenotype to the anti-inflammatory M2 phenotype, which could be reversed due to the observed *P. gingivalis*-induced decrease in *Il-10* expression [45].

*P. gingivalis* has evolved a number of mechanisms to subvert host immune defenses and suppress host inflammation [46–48]. *P. gingivalis* has been shown to promote bacterial survival via suppression of host cell apoptosis [49, 50]. *P. gingivalis* may also have indirect effects on the host immune system via dysbiotic commensals; *P. gingivalis* infection of mice fed a WD increased the abundance of *Bifidobacterium*, which has been associated with immunosuppression in mouse models [51]. This could result in disruption of inflammation that normally compensates for overfeeding by upregulating processes such as remodeling of tissues for regulation of lipid storage [52, 53]. The other major enrichment was of *Clostridium sensu stricto*, members of which are generally perceived as pathogenic [54] and as an indicator of a less healthy microbiota [33].

A well-established mechanism by which the gut bacteria can directly promote host metabolism and suppress inflammation is through the production of SCFAs such as butyrate, acetate, and propionate [12, 13]. Mouse models have demonstrated that treatment with SCFA producers such as *Lactococcus* is protective against cardiometabolic diseases by improving metabolic signaling and suppressing inflammation [14, 16]. *P. gingivalis* infection was broadly characterized by a reduction of known major SCFA producers, including *Eisenbergella, Turicibacter*, and *Lactococcus* in WD-fed mice and *Clostridium* XIVa members in CD- and WD-fed mice. Many SCFA producers are able to cross-feed on SCFAs produced by other commensal bacteria [13, 55]. Thus, the low abundance of Lachnospiraceae and Christensenellaceae observed during *P. gingivalis* infection of mice fed a WD could indicate a self-perpetuating cycle of altered SCFA and/or low butyrate production. *Christensenella* is highly health-associated and recently under development as a therapeutic agent [17]. Based on these findings and the predicted differences in SCFA production, future studies including measurement of SCFAs in the cecal contents will be critical to our understanding of the role of the microbiome in *P. gingivalis*-induced steatosis.

In mice fed a CD, acute *P. gingivalis* infection did not affect the microbiota, but 6 weeks later a substantial impact was observed, supporting a role for oral infection in dysbiosis that could promote disease over time. In the absence of differences in hepatic *Tnf*α or *Il-6* expression, *P. gingivalis*-induced insulin resistance in mice fed a CD may result from chronic low levels or intermittent exposure to intestinal inflammation or to the alteration of gut microbiota by *P. gingivalis* and the accompanying predicted reduced potential for fermentation and SCFA production based on the reduced abundance of SCFA producers observed here [56]. An increase of Bacteroidetes and in the order Bacteroidales comprised the largest taxonomic difference due to *P. gingivalis* infection of CD-fed mice. Bacteroidales correlated with both reduced insulin sensitivity and elevated expression of intestinal *Il-1*β, indicating that Bacteroidales may be a key component in the dysbiotic community promoting disease in CD-fed mice and making it a target for future therapeutic interventions.

Low alpha diversity of the gut microbiota is frequently observed in patients with NAFLD [57]. We observed reduced alpha diversity after acute *P. gingivalis* infection but these differences were no longer significant several weeks after infection. In addition, the increase in alpha diversity due to WD feeding may counterbalance the impact of *P.gingivalis* infection. Low bacterial richness correlates with insulin resistance, dyslipidemia, and adiposity in humans [58]. Fecal microbiome transplants from healthy donors to improve the insulin sensitivity of recipient patients increase not only alpha diversity but also production of butyrate by the gut microbiota [59]. Reduced production of certain SCFAs has been identified as an important pathogenic factor in NAFLD [15]. Notably, elevated CD5L has been observed in the serum of humans with cirrhotic NAFLD or hepatocellular carcinoma [60–62]. *P. gingivalis* oral infection could interfere with compensatory inflammatory mechanisms during these diseases. Thus, the ability of *P. gingivalis* to suppress *Cd5l* expression is worthy of further investigation.

In summary, our findings support a role for *P. gingivalis* in exacerbating gut dysbiosis associated with NAFLD due to a WD. We demonstrated that *P. gingivalis* infection further altered WD-induced hepatic inflammation, promoting NAFLD and glucose intolerance in steatotic mice. Furthermore, we show that *P. gingivalis* oral infection in WD-fed mice induced a sustained alteration of the gut microbiota composition and function. We identified reduced alpha diversity and reduction of SCFA producers of the gut microbiota as key features connected to NAFLD exacerbation. Collectively, findings presented here support a role for *P. gingivalis*-induced gut microbiota dysbiosis on NAFLD development. Future studies demonstrating the role of the microbiome in *P. gingivalis*-induced steatosis using fecal transplantation into germ-free mice could provide insight into the therapeutic potential of the results presented here.

## Materials and Methods

### Animals

Male C57BL/6J mice (Jackson Laboratory) of 8 weeks of age were randomly assigned to either a high-fat, high-sucrose Western diet (WD) (Teklad Global 08811; 23% fat w/w) or a refined, matched low-fat, low-glycemic index control diet (CD) (Teklad Global 120455; 6.2% fat). After 4 weeks on either diet, half the mice (*n* = 5–8) on each diet were inoculated with 100 μL of *P. gingivalis* (1 × 10^9^ CFU) suspended in vehicle (2% carboxymethylcellulose in phosphate-buffered saline), topically applied to the buccal surface of the maxillary gingiva 5 times a week for 3 weeks (Supplementary Figure 1). Vehicle-treated mice (CD-Vehicle, WD-Vehicle in Supplementary Figure 1) received the same treatment with vehicle alone. One additional group per diet was included to evaluate the effect of sham infection with vehicle, since carboxymethylcellulose is part of a standard protocol for *P. gingivalis* infection but has also been recently identified as capable of modulating the gut microbiome in mice [63]. Fecal pellets were collected 1-day post-infection. Animals were euthanized after a total of 16 weeks to allow sufficient time for NAFLD development. At sacrifice, weights were recorded for the cecum, liver, mesenteric, inguinal, and epididymal adipose tissue, and whole body (Supplementary Table 1). The ileum, half the liver, mesenteric adipose tissue, and cecal contents were flash frozen and stored at −80°C. The remaining portion of the liver was fixed overnight in 10% formalin and stored in 70% ethanol for paraffin embedding.

### Cultivation of Bacteria

*P. gingivalis* was cultured and administered as previously described [23]. Briefly, *P. gingivalis* strain 381 was grown anaerobically on blood agar plates and used to inoculate brain heart infusion broth supplemented with yeast extract, hemin, and menadione.

### Glucose and Insulin Tolerance Tests

Intraperitoneal glucose and insulin tolerance tests were performed during experimental week 13 or 14, respectively (Supplementary Figure 1). Mice were fasted for 5 h then injected intraperitoneally with either 1 g/kg of 20% glucose in PBS or 0.75 U/kg of insulin in PBS (Humulin, Eli Lilly and Company, Indianapolis IN). Blood glucose was measured at time points 0, 15, 30, 45, 90, 120 min post-injection. Glucose concentration was determined with a glucose meter (One Touch® Ultra® 2, LifeScan, Millpitas, CA). The area under the curve (AUC) (0–120 min) was calculated for each group of mice.

### RNA Preparation and Quantitative Real-Time PCR

RNA was extracted from frozen samples of the ileum, liver, and mesenteric adipose tissue using a bead-based tissue disrupter (TissueLyser II, Qiagen, Germantown, MD) and with phenol-chloroform extraction (Trizol, Invitrogen) followed by purification using a RNeasy Mini kit (Cat. no. 74104, Qiagen). RNA was made into cDNA using a High-Capacity RNA-to-cDNA™ Kit (Cat. no. 4387406, ThermoFisher Scientific, Waltham MA). Standard Taqman probes were ordered from ThermoFisher Scientific for 30 genes selected for their relevance to immune or metabolic function in the tissue of interest (probes listed in Supplemental Table 2). cDNA was probed in multiplex with the housekeeping gene *B-2-Microglobulin* (Taqman probe Mm00437762_m1) in 384-well plates using an Applied Biosystems™ QuantStudio™ 6 Flex Real-Time PCR System (ThermoFisher Scientific). Fold change was calculated using the ddCt method. Results were reported as fold relative to an average across all four primary groups in Supplementary Table 2.

### Liver Histological Analysis

Liver samples fixed in 10% formalin were embedded in paraffin. 5-μm-thick sections were processed and stained with haematoxylin and eosin (H&E). Lipid content was quantified using ImageJ software and R.

### Microbiome Analysis

Five mice per group were randomly selected for 16S rRNA gene sequencing during acute and chronic infection. Control groups (CD-None, WD-None in Supplementary Figure 1) were only sequenced during chronic infection.

Fecal samples collected the day after the final infection were stored at −80°C. DNA was extracted from samples in DNA/RNA Shield^TM^ (Zymo Research, Irvine CA) using the ZymoBIOMICS®-96 MagBead DNA Kit (Zymo Research). Library preparation and sequencing were performed by the Zymo Research Corporation (Irvine, CA). For each sample, an amplicon library of the 16S rRNA gene was constructed by performing PCR using primers flanking the variable regions 3 and 4 (*Quick*-16S™ Primer Set V3-V4; Zymo Research) using the *Quick*-16S™ NGS Library Prep Kit (Zymo Research). Positive (ZymoBIOMICS® Microbial Community Standard) and negative controls were included for quality assurance. 16S rRNA amplicon libraries were then pooled in equal molar concentration, cleaned with the Select-a-Size DNA Clean & Concentrator™ (Zymo Research), and sequenced on an Illumina® MiSeq™ with a ZymoBIOMICS® Microbial Community Standard 3 reagent kit (600 cycles, paired-end format).

Cecal contents collected at the final timepoint were stored at −80°C and processed as previously described [23]. Briefly, frozen cecal samples were homogenized using a bead-based tissue disrupter (TissueLyser II, Qiagen) followed by phenol-chloroform extraction (Trizol, Invitrogen) and ethanol precipitation. Isolated DNA was purified (AllPrep DNA/RNA Mini Kit, Qiagen). Library preparation and sequencing were performed by the Tufts University Core Facility Genomics Core (Boston, MA). For each sample, an amplicon library of the 16S rRNA gene was constructed by performing PCR using primers flanking the variable region 4, followed by a nested PCR to introduce Illumina adaptors. Positive and negative controls were included for quality assurance. 16S rRNA amplicon libraries were then pooled in equal molar concentration and sequenced with an Illumina® MiSeq™ using MiSeq V2 chemistry.

Raw data were converted to fastq format using bcl2fastq from Illumina. Unique amplicon sequences were inferred and chimeric sequences removed from raw reads using the Dada2 pipeline version 1.0.3 in R [64]. Briefly, for the 34 cecal samples collected during chronic infection, 6,523,979 reads were filtered, trimmed, and denoised using the filterAndTrim(), learnErrors(), and dada() functions down to a total of 5,994,592 sequences, 91.1% of which were successfully merged for a total of 490 non-chimeric amplicon sequence variants or ASVs (Supplementary Figure 1). Those ASVs were filtered down to 281 ASVs which were present in at least 20% of samples with a read count of more than 3 reads per sample. For the 20 fecal samples collected during acute infection, 771,769 reads were filtered, trimmed, and denoised for a total of 438,752 sequences, 99.1% of which were successfully merged. With the same criteria as above, the 392 resulting ASVs were filtered down to 171. Samples were not rarified since the smallest and largest library sizes only differed by 3-fold for cecal samples and 2-fold for fecal samples [65].

Taxonomy was assigned using the assign Taxonomy() function and the Ribosomal Database Project (RDP) [66] training set 16 (available from https://benjjneb.github.io/dada2/training.html) and a phylogenetic tree was produced using the phangorn version 2.5.5 and DECIPHER version 2.14.0 R packages.

Computational analyses and visualization of alpha and beta diversity were performed using using the phyloseq package version 1.30.0 in R and Microbiome Analyst (www.microbiomeanalyst.ca). Alpha diversity of filtered reads was assessed by the Shannon index. To measure beta diversity, the 10% of ASVs with the lowest interquartile range were removed and the remaining data was scaled using the total sum method. Beta diversity was calculated using the phylogeny-based unweighted UniFrac and weighted UniFrac distances based on the ASV distribution across samples, visualized by Principal Coordinate Analysis, and significance assessed by Permutational ANOVA (PERMANOVA) [30]. The unweighted UniFrac distance matrices measures ASV presence, while the weighted UniFrac distance matrices account for ASV abundance. Taxonomic abundance calculated in R. To elucidate community biomarkers of treatment effects, differential abundance of taxa across experimental groups was assessed using Linear discriminant analysis (LDA) of effect size (LEfSe) in the online Galaxy workflow framework (http://huttenhower.sph.harvard.edu/galaxy/).

Microbial gene content was inferred from ASV abundance using Phylogenetic investigation of communities by reconstruction of unobserved states 2 (PICRUSt2) (https://github.com/picrust/picrust2; v2.3.2-b) [67]. PICRUSt2 is a significant expansion of PICRUSt with a reference genomes database over ten times the size and provides MetaCyc pathway predictions comparable with typical shotgun metagenomics datasets [67]. the DESeq2 package in R with an adjusted *p*-value cutoff for significance of 0.05. Differentially abundant inferred pathways were visualized in R. Despite the improvements in PICRUSt2, a major limitation of PICRUSt inferences is that it depends on accurate gene annotations. Previous research has shown that microbial gene annotations are notoriously inaccurate, making biological interpretations of microbiome community function uncertain [68]. In addition, these genes may not be transcribed or translated, limiting the impact of their annotated function. Conclusions about microbiome function derived either from metagenomics or from PICRUSt should be treated as hypotheses that require further in-depth validation through functional assays.

### Statistical Analyses

Student's *t*-test was applied to compare two groups. One-way analysis of variance followed by a *t*-test with Bonferroni correction or a two-way, repeated measures analysis of variance followed by a *t*-test with Tukey correction were performed for multiple group comparisons using RStudio or GraphPad PRISM. Graphical inspection using a Q-Q plot was employed to test normality. Data that violated normality was log-transformed. R, GraphPad PRISM 8, and microbiomeanalyst.ca were used for statistics and graphics. Unless otherwise noted, an α of 0.05 was considered to reject null hypothesis.

## Data Availability Statement

The data presented in the study are deposited in the EMBL Nucleotide Sequence Database (ENA) (https://www.ebi.ac.uk/ena/browser/home), accession number PRJEB48939, and secondary accession number ERP133380. The data were submitted on November 23, 2021 and the release date is currently set for January 31, 2022.

## Ethics Statement

The animal study was reviewed and approved by Tufts University Institutional Animal Care and Use Committee.

## Author Contributions

AS: study concept and design, data acquisition, data analysis and interpretation, and manuscript production. CK: study concept and design, data acquisition, and manuscript preparation. CG: study concept and design, data interpretation, and manuscript preparation. All authors contributed to the article and approved the submitted version.

## Funding

AS was supported by the National Institutes of Health with a grant from the T32 DK062032.

## Conflict of Interest

The authors declare that the research was conducted in the absence of any commercial or financial relationships that could be construed as a potential conflict of interest.

## Publisher's Note

All claims expressed in this article are solely those of the authors and do not necessarily represent those of their affiliated organizations, or those of the publisher, the editors and the reviewers. Any product that may be evaluated in this article, or claim that may be made by its manufacturer, is not guaranteed or endorsed by the publisher.
