# Supplementary material for: Diet-Induced Non-alcoholic Fatty Liver Disease and Associated Gut Dysbiosis Are Exacerbated by Oral Infection
Source: Front Oral Health. 2022 Jan 24;2:784448. doi: 10.3389/froh.2021.784448 (PMC8820505; doi:10.3389/froh.2021.784448)
Supplement: Supplementary file 1 [file Data_Sheet_1.PDF]

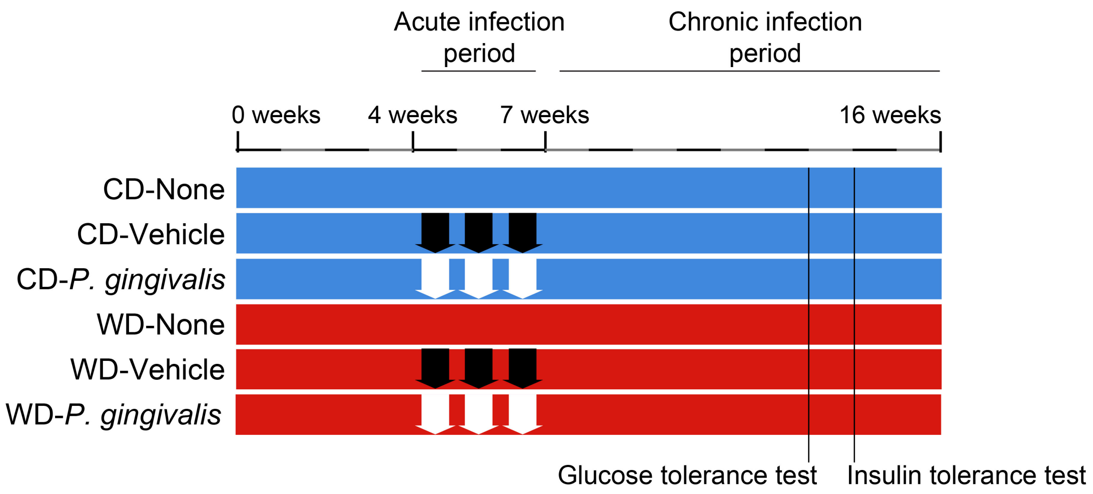

Supplementary Figure S1. Experimental design. Mice received either a Western diet (WD) or a control diet (CD) for a total of sixteen weeks. Within each diet, mice were either sham-infected with vehicle (carboxymethylcellulose) (CD-Vehicle, WD-Vehicle) or infected with 1 x 10^6 CFUs of *P. gingivalis* suspended in vehicle (CD-*P. gingivalis*, WD-*P. gingivalis*) five times per week for three weeks. Fecal pellets were collected 1-day post-infection. To examine the effect of vehicle, two additional control groups were not sham-infected (CD-None and WD-None). Glucose and insulin tolerance tests were performed at weeks 13 and 14, respectively. All mice were sacrificed at 16 weeks. At this time, the liver was analyzed for NAFLD by histology. Gene expression was measured in the liver, adipose tissue, and ileum. 16S sequencing was performed on the 1-day post-infection fecal pellets and the final cecal contents.

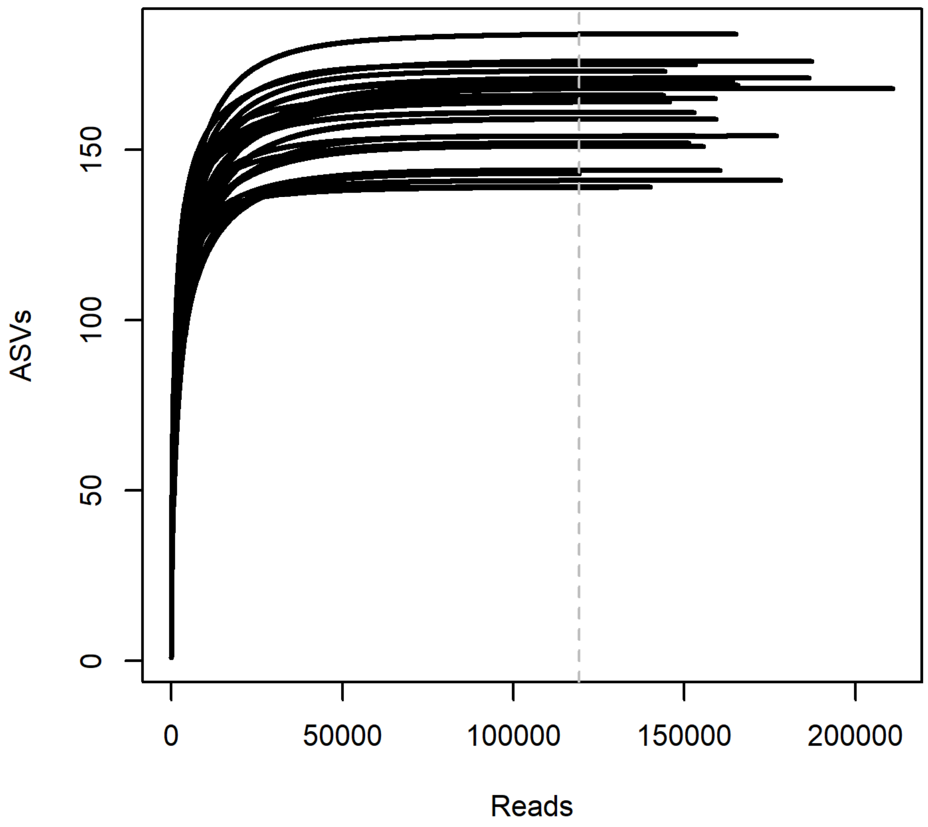

Supplementary Figure S2. Chronic *P. gingivalis* infection read library size and rarefaction curves. Rarefaction curves based on unfiltered libraries made from the results of cecal 16S sequencing. Lines represent the mean and error bars represent standard deviations. The analysis was performed on a randomly selected subsets over 100 iterations.

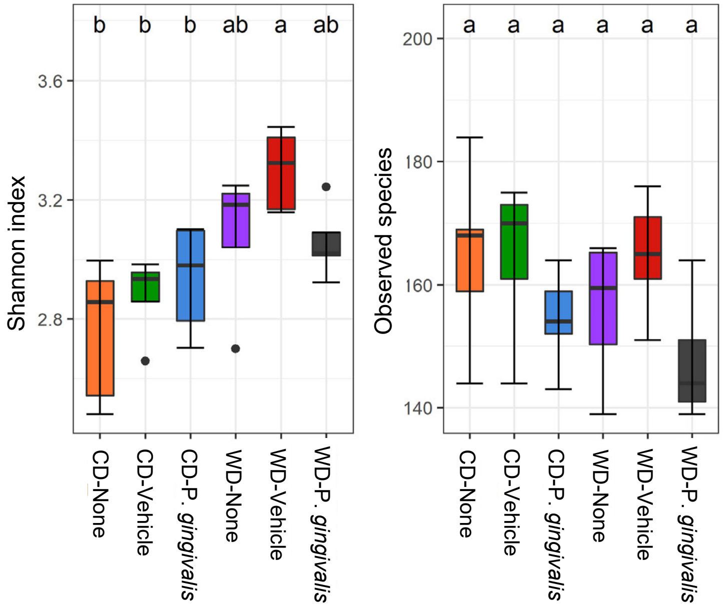

Supplementary Figure S3. A Western diet increases the evenness of the cecal microbiota. Results of 16S sequencing of the cecal microbiome during chronic infection with *P. gingivalis* or treatment with vehicle compared to control groups that received neither (CD-None, WD-None). Significance of Shannon index and ASV counts per sample tested with a one-way ANOVA and Tukey's Honestly Significant Difference post-hoc test. Groups with the different letters are significantly different.

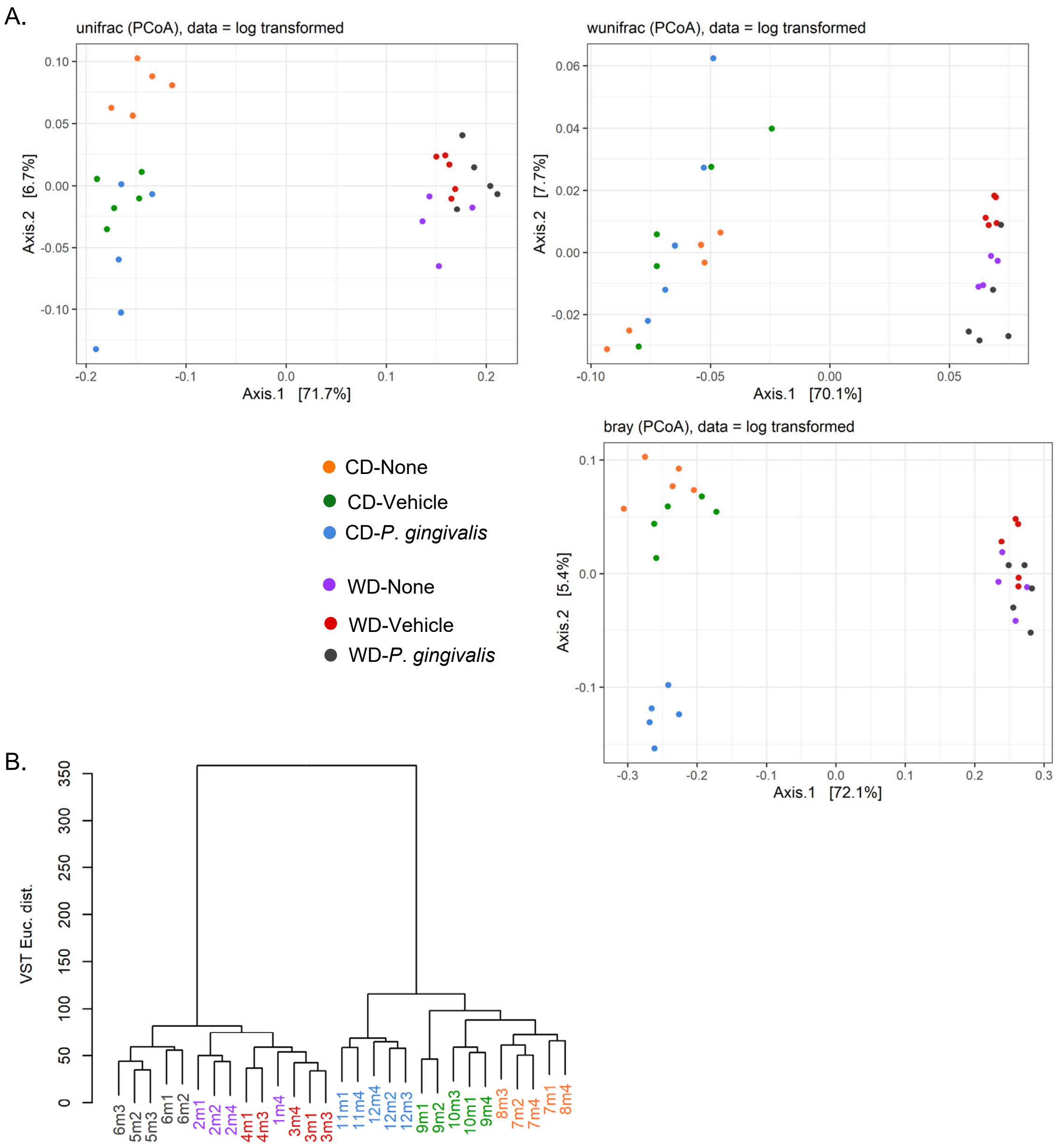

Supplementary Figure S4. A Western diet and chronic *P. gingivalis* infection both alter the beta diversity of the cecal microbiota. Results of 16S sequencing of the cecal microbiome during chronic infection with *P. gingivalis* or treatment with vehicle compared to control groups that received neither (CD-None, WD-None). A) Unweighted UniFrac, weighted UniFrac, and Bray-Curtis indices of beta diversity with significance measured by PERMANOVA. Unweighted Unifrac: [PERMANOVA] F-value: 29.398; R-squared: 0.83999; p-value < 0.001. Weighted Unifrac: [PERMANOVA] F-value: 5.1198; R-squared: 0.4776; p-value < 0.001. Data are log-transformed and plotted by Principal Components Analysis (PCoA). B) Dendrogram of samples hierarchically clustered by beta diversity as measured by Euclidean distance of sample relatedness. Orange = uninfected mice fed a CD, green = vehicle-treated mice fed a CD, blue = *P. gingivalis*-infected mice fed a CD; purple = uninfected mice fed a WD, red = vehicle-treated mice fed a WD, black = *P. gingivalis*-infected mice fed a WD.

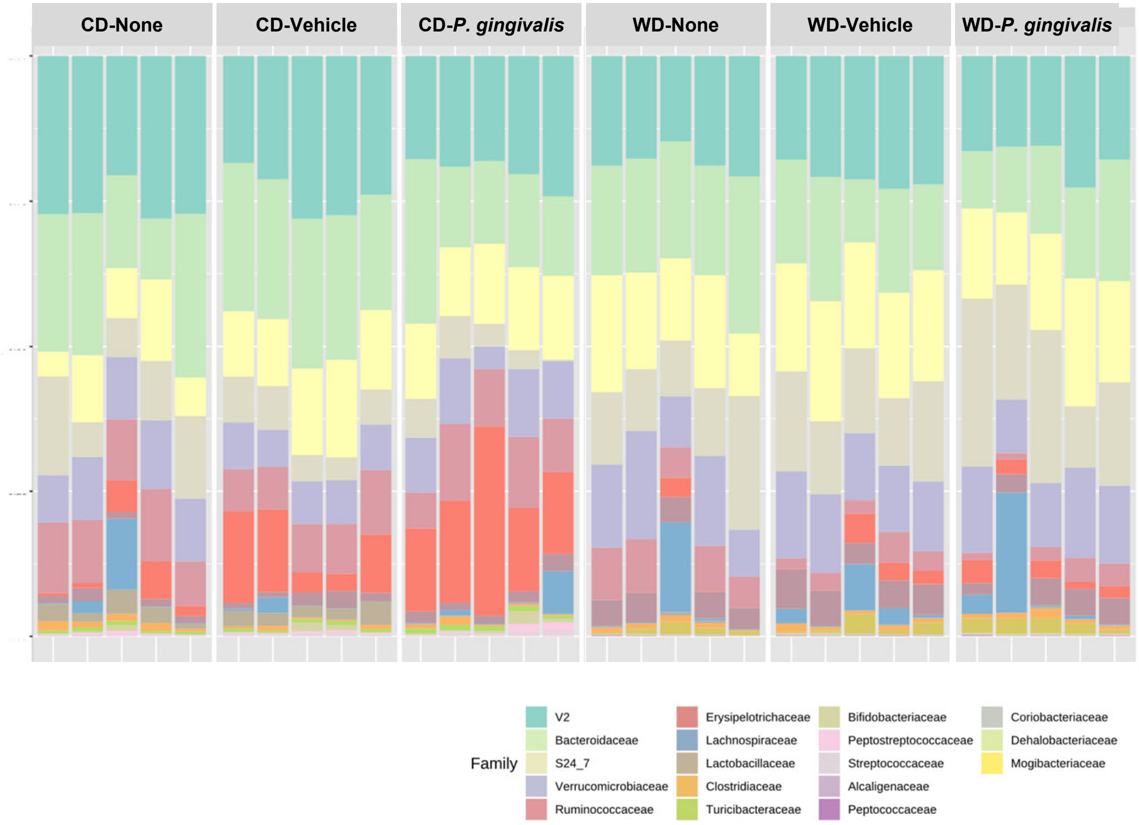

Supplementary Figure S5. Chronic *P. gingivalis* has a more profound impact on the taxonomic abundance of mice fed a control diet than a Western diet. Relative taxonomic abundance at family level from 16S sequencing of the cecal microbiome during chronic infection. n=5/group.

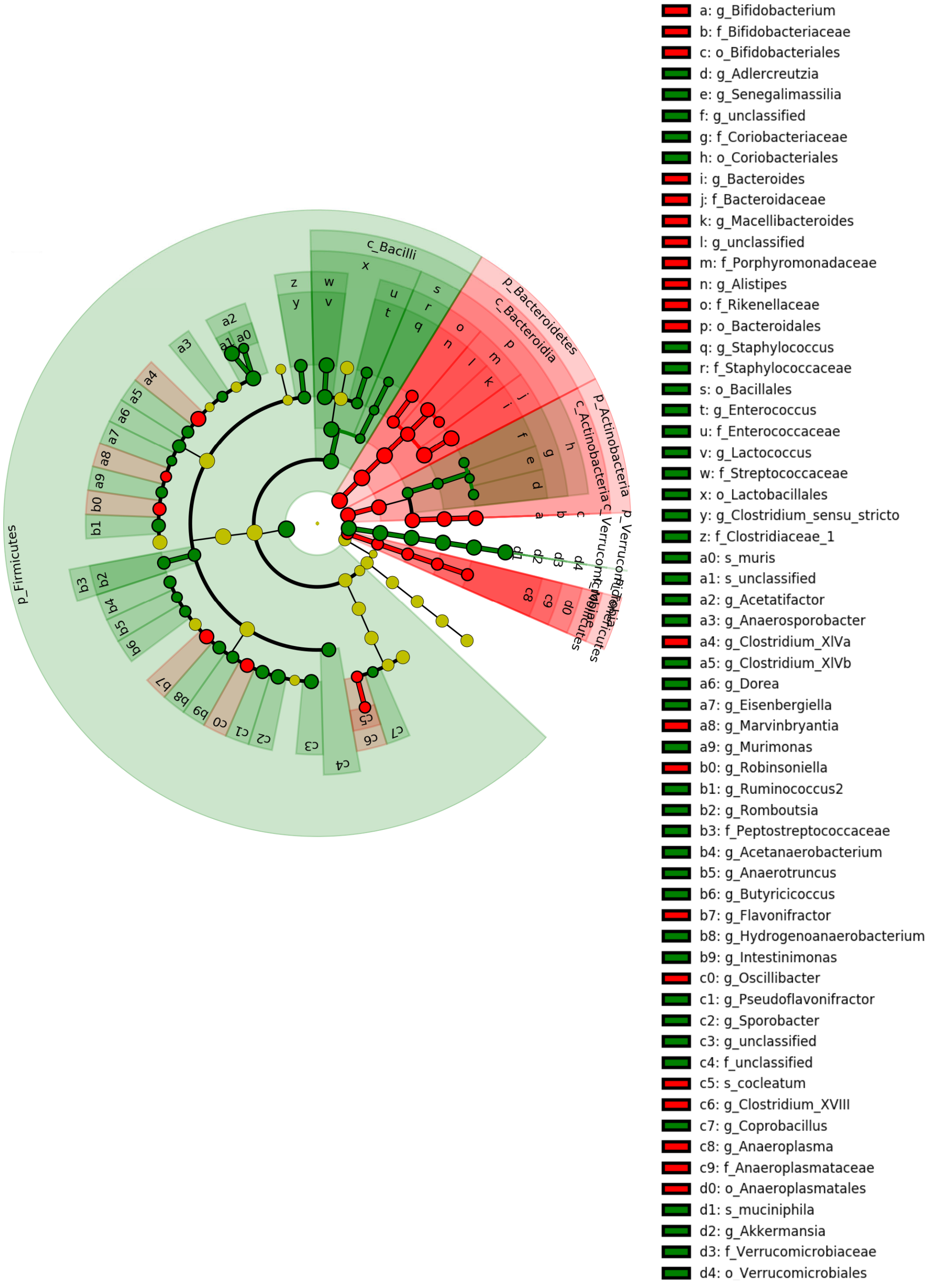

Supplementary Figure S6. Effect of WD feeding during chronic infection period. Linear discriminant analysis (LDA) effect size (LEfSe) analysis of predicted taxonomy from 16S sequencing of the cecal microbiota comparing vehicle-treated mice fed a WD with vehicle-treated mice fed a CD. LDA > 2.0, α < 0.05. Green indicates increased abundance while red indicates reduced abundance in response to WD feeding.

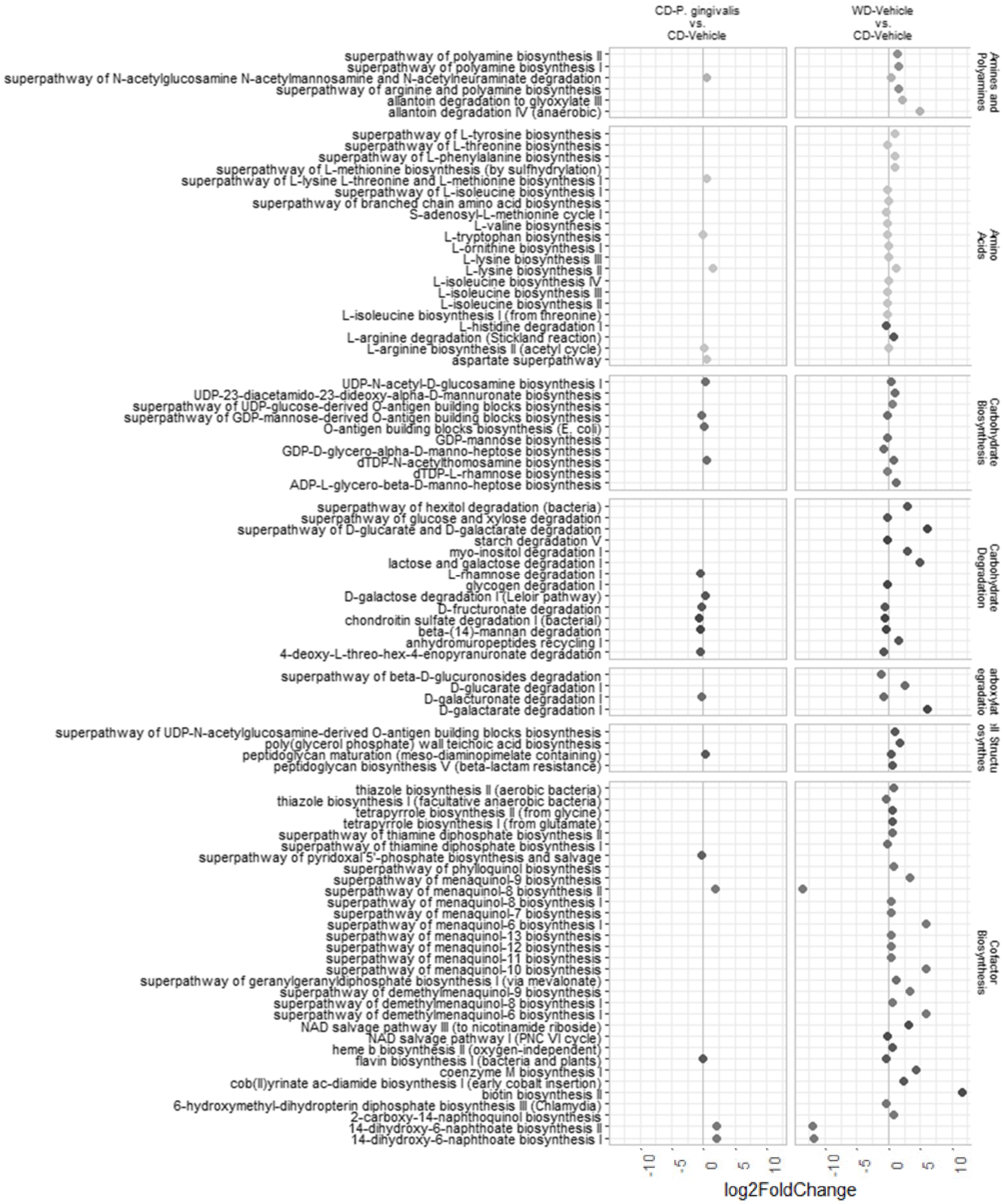

Supplementary Figure S7. Predicted pathway enrichment during chronic infection. Predicted pathways expected to be differentially abundant due to *P. gingivalis* infection of mice fed a CD or the effect of a WD alone. No significant differences were observed between *P. gingivalis*-infected, WD-fed mice and vehicle-treated, WD-fed mice. Significance assessed by DESeq2 with an adjusted p-value < 0.01.

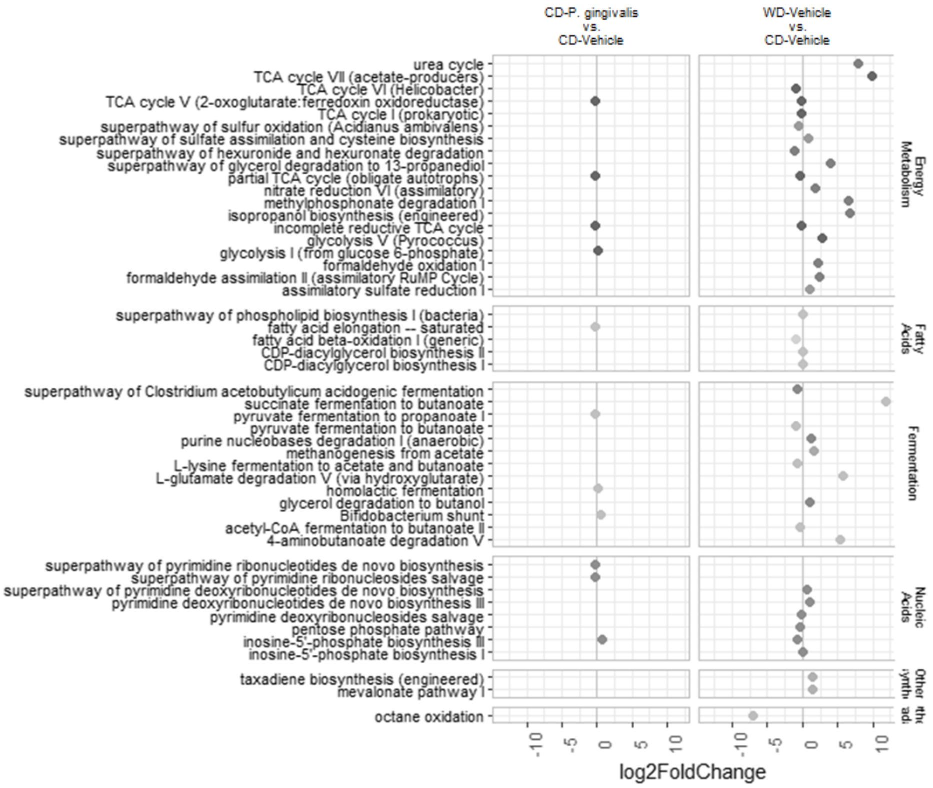

Supplementary Figure S7 (continued). Predicted pathway enrichment during chronic infection. Predicted pathways expected to be differentially abundant due to *P. gingivalis* infection of mice fed a CD or the effect of a WD alone. No significant differences were observed between *P. gingivalis*-infected, WD-fed mice and vehicle-treated, WD-fed mice. Significance assessed by DESeq2 with an adjusted p-value < 0.01.

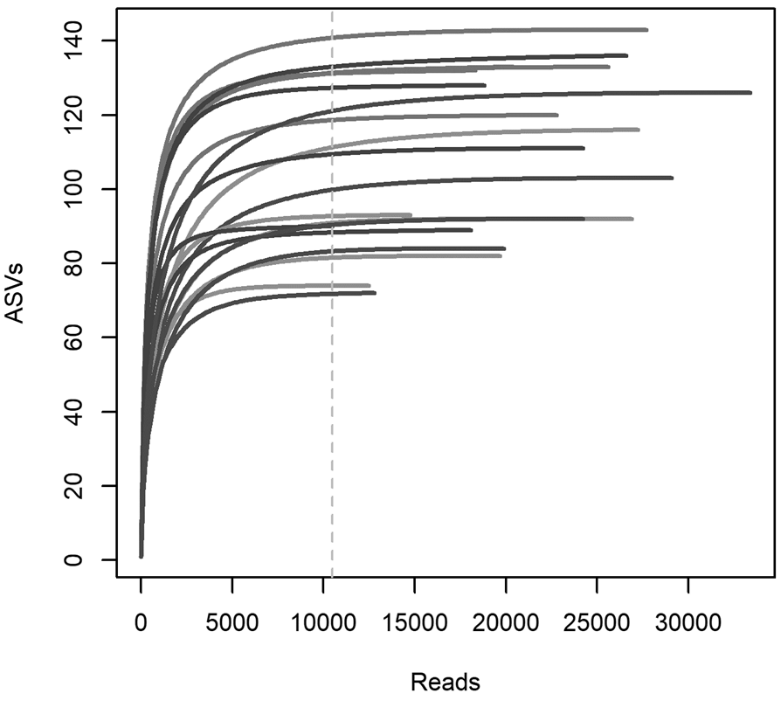

Supplementary Figure S8. Acute *P. gingivalis* infection read library size and rarefaction curves. Rarefaction curves based on unfiltered libraries made from the results of fecal 16S sequencing. Lines represent the mean and error bars represent standard deviations. The analysis was performed on a randomly selected subsets over 100 iterations.

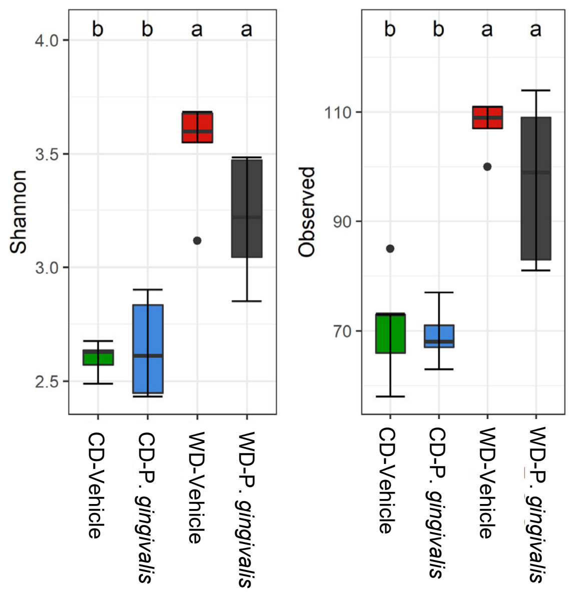

Supplementary Figure S9. Results of 16S sequencing of the cecal microbiome during acute infection with *P. gingivalis* or sham-infection. Significance of Shannon index and ASV counts per sample tested with a one-way ANOVA and Tukey's Honestly Significant Difference post-hoc test. Groups with the different letters are significantly different.

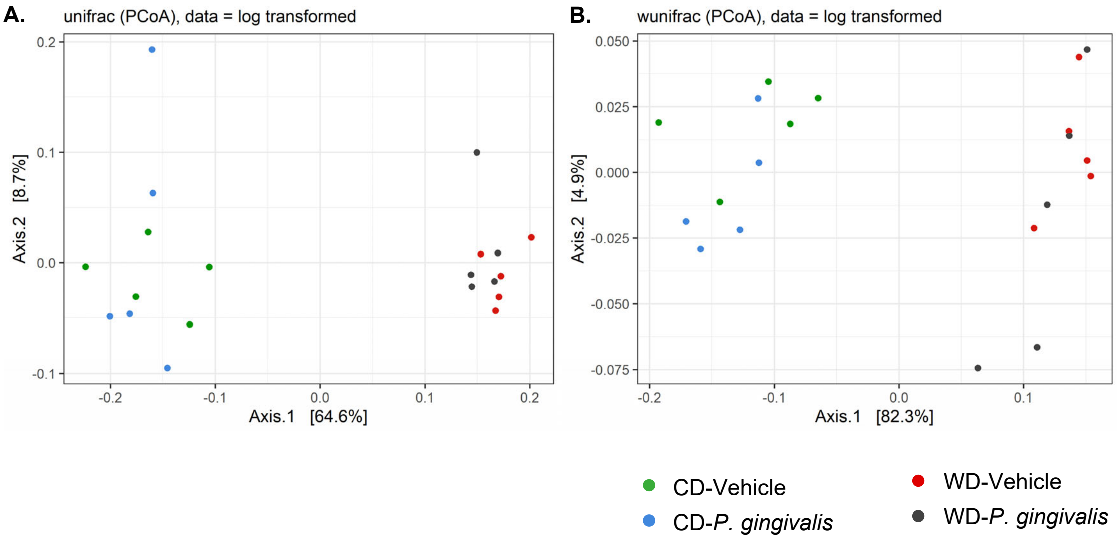

Supplementary Figure S10. Acute *P. gingivalis* infection alters relative abundance of the gut microbiome of mice fed either a CD or a WD. A) Unweighted UniFrac with significance measured by PERMANOVA (all groups: R^2 = 0.7442, p < 0.001; *P. gingivalis* vs.vehicle-treated mice fed a WD: R^2 = 0.2068, p = 0.063; *P. gingivalis* vs. vehicle-treated mice fed a CD: R^2 =0.1598, p = 0.107). B) Weighted UniFrac with significance measured by PERMANOVA (all groups: R^2 = 0.7384, p < 0.001; *P. gingivalis* vs. vehicle-treated mice fed a WD: R^2 =0.0647, p = 0.68; *P. gingivalis* vs. vehicle-treated mice fed a CD: R^2 =0.1524, p = 0.228).

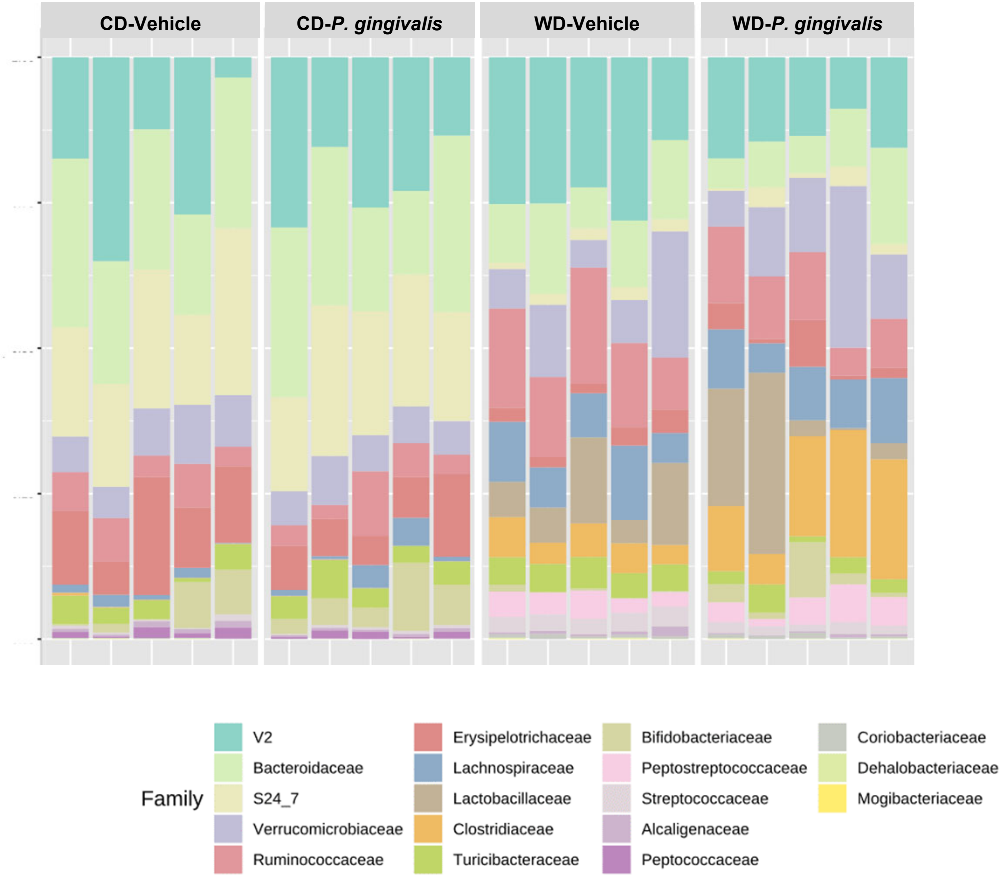

Supplementary Figure S11. Acute *P. gingivalis* infection alters relative abundance of the gut microbiome of mice fed either a CD or a WD. Relative taxonomic abundance at the family level from 16S sequencing of the fecal microbiome during acute infection. n=5/group.

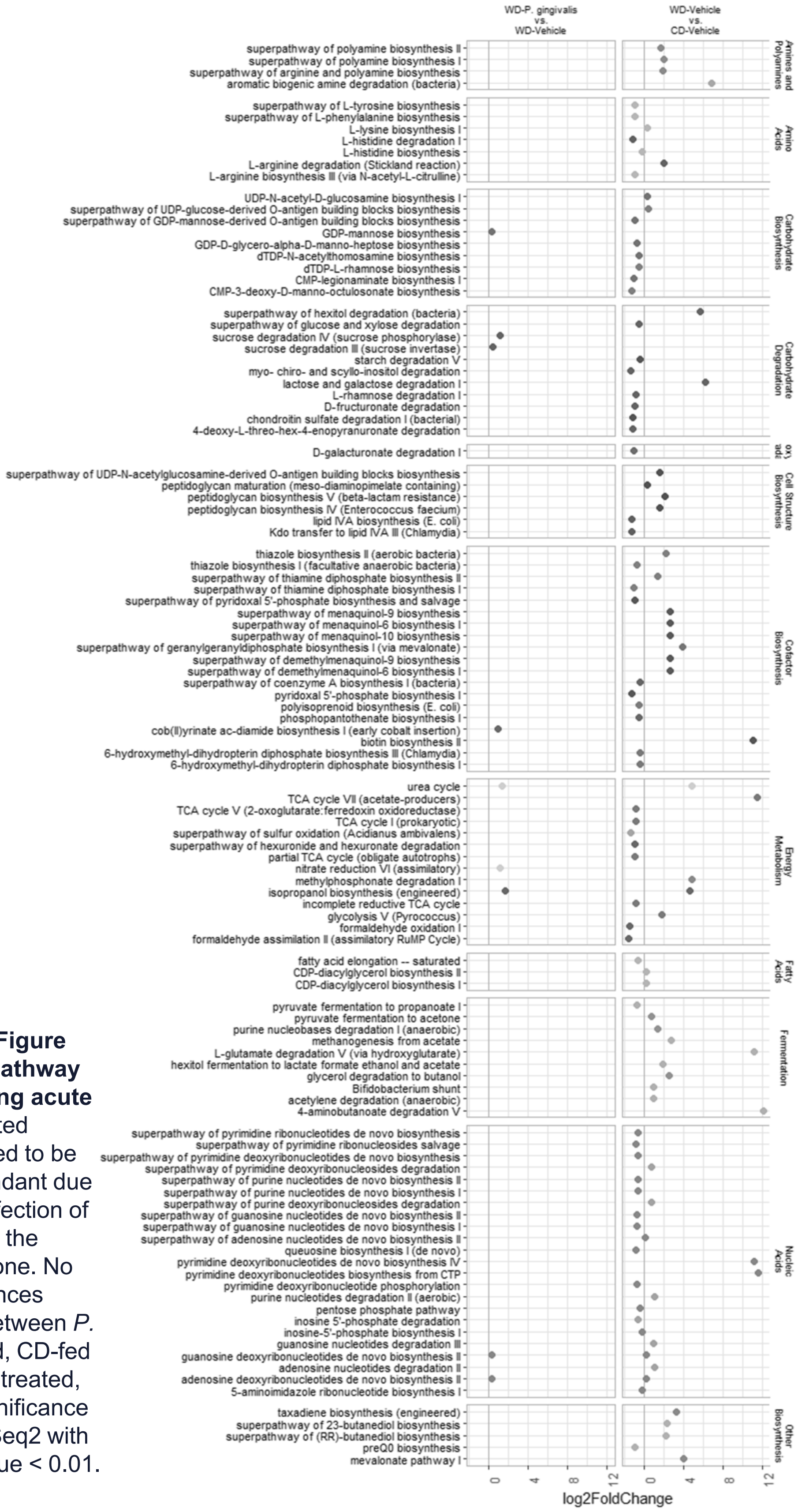

Supplementary Figure S12. Predicted pathway enrichment during acute infection. Predicted pathways expected to be differentially abundant due to *P. gingivalis* infection of mice fed a WD or the effect of a WD alone. No significant differences were observed between *P. gingivalis*-infected, CD-fed mice and vehicle-treated, CD-fed mice. Significance assessed by DESeq2 with an adjusted p-value < 0.01.

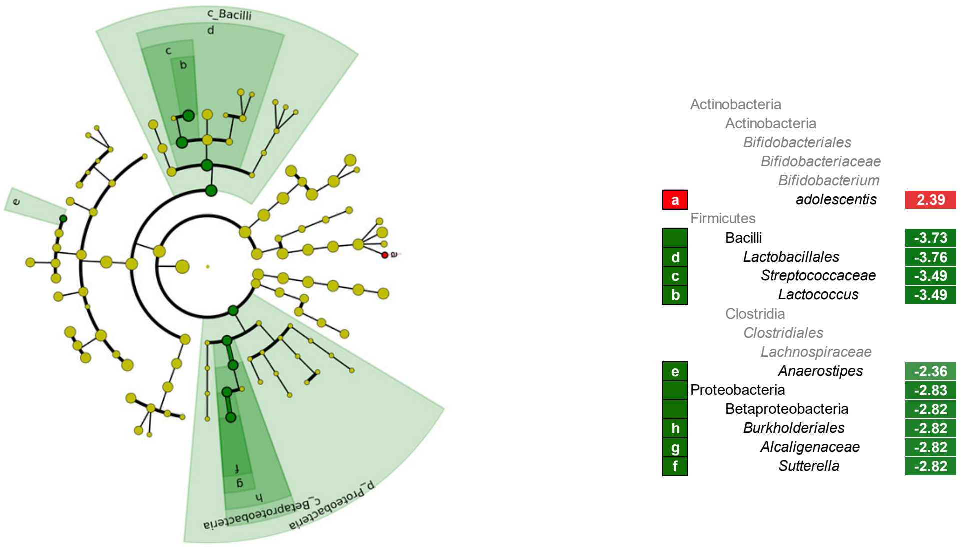

Supplementary Figure S13. Treatment with carboxymethylcellulose (vehicle) mildly influences the cecal microbiome during the chronic infection period. Linear discriminant analysis (LDA) effect size (LEfSe) analysis of predicted taxonomy from 16S sequencing of the cecal microbiota comparing vehicle-treated mice fed a CD with uninfected mice fed a CD. LDA > 2.0, α < 0.05. Green indicates increased abundance while red indicates reduced abundance in response to treatment with vehicle.

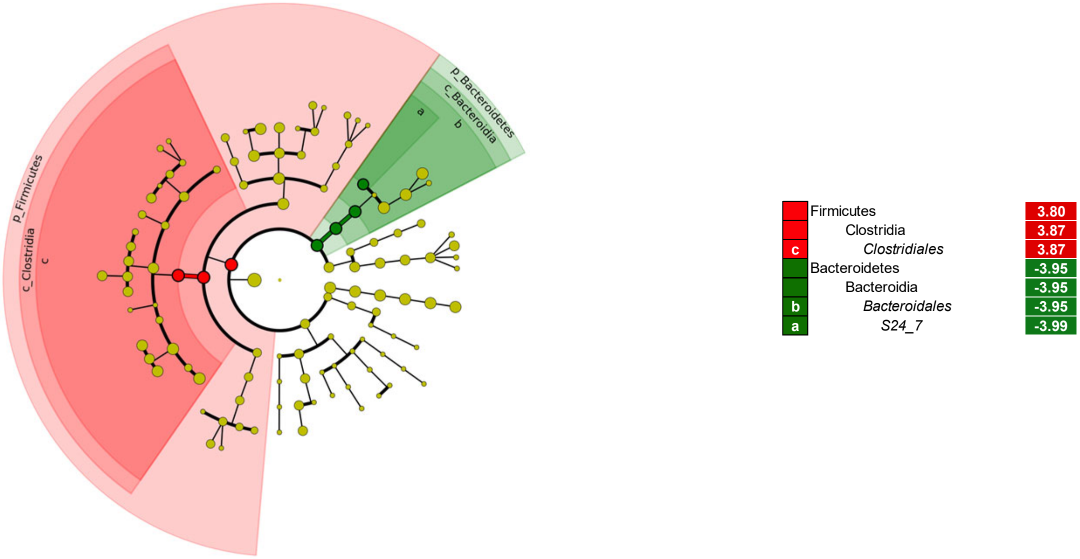

Supplementary Figure S14. Treatment with carboxymethylcellulose (vehicle) mildly influences the fecal microbiome during an acute infection period. Linear discriminant analysis (LDA) effect size (LEfSe) analysis of predicted taxonomy from 16S sequencing of the fecal microbiota comparing vehicle-treated mice fed a CD with uninfected mice fed a CD. LDA > 2.0, α < 0.05. Green indicates increased abundance while red indicates reduced abundance in response to treatment with vehicle.
